# A Hybrid Modeling Technique of Epidemic Outbreaks with Application to COVID-19 Dynamics in West Africa

**DOI:** 10.3390/biology10050365

**Published:** 2021-04-23

**Authors:** Chénangnon Frédéric Tovissodé, Jonas Têlé Doumatè, Romain Glèlè Kakaï

**Affiliations:** 1 Laboratoire de Biomathématiques et d’Estimations Forestières, Université d’Abomey-Calavi, Abomey-Calavi, Benin; chenangnon@gmail.com (C.F.T.); jonas.doumate@fast.uac.bj (J.T.D.); 2 Faculté des Sciences et Techniques, Université d’Abomey-Calavi, Abomey-Calavi, Benin

**Keywords:** growth model, epidemic latency period, reproduction number, West Africa

## Abstract

**Simple Summary:**

The intrinsic dynamics of the propagation of a disease changes along an epidemic course, especially for long lasting epidemics such as the COVID-19. Indeed, the natural evolution of the pathogen and countermeasures such as quarantining, lockdown, social distancing and vaccination modify the transmission dynamics of the disease. With a view to match these theoretical changes to potential changes in observed epidemiological data, we designed a hybrid modeling framework where we integrated: (1) two growth curves for daily reported positive cases, differentiating the early epidemic phase and a second phase with a potentially different dynamics; (2) two logistic regression models for daily recoveries and deaths; and (3) a SIQR (Susceptible, Infective, Quarantined, Recovered) mechanistic model to provide an overview of the dynamics of the disease in the target population. This joint modeling approach allows explicit analytical expressions for the different compartments of the SIQR model, circumventing common identifiability issues in such models. The changes in the disease transmission pattern can be subjected to countermeasures so as to assess their effectiveness along time. For illustrative purposes, we applied the approach to COVID-19 data from West Africa. It turned out that the first imported COVID-19 case(s) in West Africa likely entered the region between 28 January and 7 February 2020. Moreover, the first measures implemented by West African authorities impacted the dynamics of the disease one month after the outbreak.

**Abstract:**

The widely used logistic model for epidemic case reporting data may be either restrictive or unrealistic in presence of containment measures when implemented after an epidemic outbreak. For flexibility in epidemic case reporting data modeling, we combined an exponential growth curve for the early epidemic phase with a flexible growth curve to account for the potential change in growth pattern after implementation of containment measures. We also fitted logistic regression models to recoveries and deaths from the confirmed positive cases. In addition, the growth curves were integrated into a SIQR (Susceptible, Infective, Quarantined, Recovered) model framework to provide an overview on the modeled epidemic wave. We focused on the estimation of: (1) the delay between the appearance of the first infectious case in the population and the outbreak (“epidemic latency period”); (2) the duration of the exponential growth phase; (3) the basic and the time-varying reproduction numbers; and (4) the peaks (time and size) in confirmed positive cases, active cases and new infections. The application of this approach to COVID-19 data from West Africa allowed discussion on the effectiveness of some containment measures implemented across the region.

## 1. Introduction

The ravages of the COVID-19 pandemic has deepened the need for mathematical and statistical tools to understand the dynamics of epidemics across the world. Simple mathematical models of infectious diseases are useful for providing insight into epidemic trajectories and disease dynamics [1,2,3]. However, applications should target complex but parsimonious models which make realistic assumptions and let the observed data drive estimations.

There are two common approaches to epidemiological modeling: phenomenological models and mechanistic models (e.g., compartmental models). On the one hand, phenomenological models use an empirical approach based on growth curve fitting (e.g., by nonlinear least squares [4] or by maximum likelihood [5]) to describe the temporal progression of case counts (e.g., daily confirmed positive cases). In this regard, the logistic bell curve has been widely used for various epidemic data, but it lacks flexibility for epidemics whose data exibits asymmetry or varying growth patterns [4,6,7]. With a view to allow flexibility, Tovissodé et al. [5] considered the generic growth curve of Turner et al. [8] with application to COVID-19 data. This approach concedes the simple logistic curve when it is supported by the observed data, but offers the possibility to fit various flexible growth models such as the generalized logistic model [9,10], the hyperlogistic model [8,11], the hyper-Gompertz [8] and the Gompertz curves [12,13]. However, to be realistic, models for epidemic data should be able to account for the potential effect of containment measures when implemented after an epidemic outbreak. In a target population undergoing an epidemic wave, the number of infective individuals may be assumed to follow an exponential growth in the early epidemic phase where no containment measures were implemented or the implemented measures were not yet effective [14]. In this case, the variation of the number of infective individuals is expected to shift to a sub-exponential growth resulting from negative feedbacks due to a decrease in the probability that an infectious individual meets a susceptible individual [6] or effects of the containment measures, if any. The major advantage of the phenomenological modeling approach is its simplicity while allowing the estimation of various quantities of interest to understand an epidemic, e.g., the “epidemic latency period” defined as the delays between the appearance of the first infectious case in the population (“patient zero”) and the outbreak [14] and epidemic peak time and size, and the forecast of future incidence. The main limitation of phenomenological models is the inability to inform on the transmission process (new infections) and the removal processes (recovery and death) of an epidemic. As a result, phenomenological modeling lacks the ability to assess the effects of control interventions.

On the other hand, and contrary to phenomenological models, mechanistic models structure the population under study into different epidemiological states [4] and allow assessing the effects of control interventions on the population and disease dynamics. For instance, the effect of various control measures (e.g., contact limitation, detection and diagnosis) on COVID-19 transmission has been assessed using the Susceptibles–Exposed–Infectives–Recovered (SEIR) model and its variants [15,16,17]. However, because only a few epidemiological states can be observed, mechanistic models often face an identifiability issue in the estimation of model parameters [18,19,20]. In addition, there is generally no closed form solutions to the differential equations describing the considered epidemiological states. As a consequence, the estimation of compartmental models often relies on numerical approximations which make fitting procedures (e.g., nonlinear least squares or Bayesian estimation) computationally intensive and may introduce high-order errors in both estimates and forecasts [21]. Moreover, some quantities of high interest to understand epidemic outbreaks, which are readily available from a growth model including the epidemic latency period, are hard to derive under compartmental models.

This study proposes a hybrid framework to combine the advantages of phenomenological and mechanistic models while circumventing some of the limits of the two approaches. We focus on epidemic waves managed with at least an isolation measure for all identified infectives, as for the COVID-19 pandemic in nearly all the world. The objective of this work is to provide a quantitative framework in which epidemiologists can identify, from a large family of models, the parsimonious model that explains patterns in an observed dataset, and then assess hypotheses on the potential course of related but unobservable processes of interest. Specifically, we modeled confirmed positive cases using a combination of the exponential growth curve for the initial epidemic phase and the generic growth curve [8] after this initial phase. This development allows the estimation of the duration of the exponential growth phase and the theoretical time and size of the peak of new positive cases. Secondly, we modeled removal (recovery and death) from identified positive cases as binary processes using two logistic regression models to monitor the evolution and the peak (time and size) of the actives among detected cases. Finally, to provide an overall view for a target epidemic, we integrated the growth curve and the logistic regression removal rates into a mechanistic SIQR model frame [22] in which the population is structured in Susceptibles, Infectives, Quarantined (identified actives cases) and Recovered individuals. The result is a mechanistic model in which the sizes of the different states (compartments) have closed form expressions. This allows inference on various epidemiological parameters such as the delay between the appearance of the first infectious case in the population (“patient zero”) and the outbreak (“epidemic latency period”), the reproduction number, the unobservable new infections per unit time as well as the proportion of the target population immunized against the pathogen of the target disease.

In addition to the estimates (with quantified uncertainty) for common epidemiological parameters, the proposed hybrid modeling framework extracts from the observed data and demographic rates, the evolution along the epidemic course of the key parameter to summarize the dynamics of an epidemic: the reproduction number. The changes in this parameter can thus be confronted to control measures promoted/enforced by public health authorities and governments. For illustrative purpose, we used the developed modeling framework: (i) to model COVID-19 case reporting data (daily PCR-confirmed positives, recoveries and deaths) from Western Africa (28 February to 31 August 2020); and (ii) to evaluate the transmission pattern of the disease in the region during the considered period. The results were used to discuss the effectiveness of some containment measures implemented by governments across the region.

## 2. The Hybrid Modeling Framework

In this section, we describe the three sub-models integrated into the proposed modeling framework, namely, the growth model, the logistic removal rates and the Susceptible–Infective–Quarantined–Recovered (SIQR) mechanistic model.

### 2.1. Mixture of Growth Models for Detected Cases

We assume that the cumulative number $C_{t}$ of reported cases, as a function of time *t*, has the form

(1) $$\begin{array}{ccc} C_{t} & = & \left\{\begin{array}{cc} 0\; & \;\mathrm{if}\;\;t\leq 0\\ e^{\omega_{0}\left(t-\tau_{0}\right)}\; & \;\;\;\;\;\;\mathrm{if}\;\;0<t\leq t_{e}\\ \xi +\varphi_{t}\; & \;\;\mathrm{if}\;\;t>t_{e} \end{array}\right. \end{array}$$

where $t_{e}>0$ is the duration from outbreak to the end of the exponential growth phase,

(2) $$\begin{array}{c} \varphi_{t}=\Omega {\left(1+u_{t}\right)}^{-1/\nu } \end{array}$$

is the generic growth model [8] with $u_{t}={\left[1+\omega \nu \rho (t-\tau )\right]}^{-1/\rho }$, Ω>0 is a constant such that the ultimate epidemic size (detected) is ξ+Ω, ω>0 is the “intrinsic” growth rate constant for the sub-exponential growth phase, ν>0 is a growth acceleration parameter, ρ ($-1<\rho <\nu^{-1}$) is a shape parameter controlling the skewness of the growth curve during the sub-exponential epidemic phase (see Appendix A.1 for restriction related details) and τ is a constant of integration determined by the initial conditions of the epidemic. The generic growth curve $\varphi_{t}$ specified for $t>t_{e}$ encompasses many special or limiting cases including the Bertalanffy–Richards (ρ→0), hyper-Gompertz (ν→0 while $\omega \nu^{1+\rho }\to \tilde{\omega }$ with $\tilde{\omega }$ constant), Gompertz (ν→0, ρ→0 while $\omega \nu \to \tilde{\omega }$), hyper-logistic (ν=1) and logistic (ν=1 and ρ→0) growth models [8] (see Appendix A.1 for details). The parameter $\omega_{0}>0$ in (1) is the exponential growth rate for the early epidemic phase and $\tau_{0}\in R$ determines the growth rate at t=0. The constants $\omega_{0}$ and $\tau_{0}$ are set such that the first derivative ${\dot{C}}_{t}$ and the second derivative ${\ddot{C}}_{t}$ of $C_{t}$ with respect to *t* are smooth at $t=t_{e}$ (i.e., at the end of the exponential growth phase). Specifically,

(3) $$\begin{array}{ccc} \omega_{0} & = & {\ddot{\varphi }}_{e} \end{array}$$

(4) $$\begin{array}{ccc} \tau_{0} & = & t_{e}+\frac{\log\omega_{0}-\log{\dot{\varphi }}_{e}}{\omega_{0}} \end{array}$$

where ${\dot{\varphi }}_{e}={\dot{\varphi }}_{t_{e}}$ and ${\ddot{\varphi }}_{e}={\ddot{\varphi }}_{t_{e}}$; ${\dot{\varphi }}_{t}$ and ${\ddot{\varphi }}_{t}$ are, respectively, the first and second derivatives of $\varphi_{t}$ (see Appendix A.1 for details); and (4) follows from setting $\omega_{0}e^{\omega_{0}\left(t_{e}-\tau_{0}\right)}={\dot{\varphi }}_{e}$. Furthermore, the real constant ξ in (1) ensures that $C_{t}$ does not jump at $t=t_{e}$. In other words, ξ is given by $\xi =e^{\omega_{0}\left(t_{e}-\tau_{0}\right)}-\varphi_{e}$ (with $\varphi_{e}=\varphi_{t_{e}}$) which by (4) simplifies to

(5) $$\begin{array}{c} \xi =\frac{\dot{\varphi_{e}}}{\omega_{0}}-\varphi_{e}. \end{array}$$

In (1), the time (in e.g., days, weeks or months) of the first identified cases corresponds to t=1. In other words, to match (1) to the observed data, $C_{1}$ is identified to the number of cases reported in the time interval (0,1], $C_{2}$ is the number of cases reported in the time interval (0,2], etc. If Ω→∞ and νρ→0, the curve $C_{t}$ converges to an exponential growth curve with rate $\omega_{0}$. However, this scenario can be ruled out since the size of any target population is finite and so is Ω. In practice, the exponential growth is prevented by negative feedbacks which decrease the probability that an infectious individual and a susceptible individual meet and have an adequate contact (i.e., contact sufficient for transmission). For instance, the growth of the infectives is naturally continuously lowered by the increasing fraction of the population constituted by individuals who recovered and become less susceptible (temporarily or permanently immune) to the infection [6]. To prevent the exponential growth of the infectives, control measures such as quarantining and lockdown reduce the probability of contact between susceptible and infectious individuals, whereas some other measures such as social distancing and wearing a face mask reduce the likelihood of transmission whenever contacts happen.

The specification of the growth model in (1) to an epidemic thus implies that the growth rate ${\dot{C}}_{t}$, i.e., the number of new cases reported per unit time given by

(6) $$\begin{array}{c} {\dot{C}}_{t}=\left\{\begin{array}{cc} \;\omega_{0}e^{\omega_{0}\left(t-\tau_{0}\right)} & \;\;\;\;\;\mathrm{if}\;\;0\leq t\leq t_{e}\\ {\dot{\varphi }}_{t} & \mathrm{if}\;\;t>t_{e} \end{array}\right. \end{array}$$

with ${\dot{\varphi }}_{t}$ defined in Appendix A.1, will peak and then fall toward zero case per unit time. The peak occurs at a time $t_{p}>t_{e}$ when the growth acceleration ${\ddot{C}}_{t}$ given by,

(7) $$\begin{array}{c} {\ddot{C}}_{t}=\left\{\begin{array}{cc} \;\omega_{0}^{2}e^{\omega_{0}\left(t-\tau_{0}\right)} & \;\;\;\;\;\mathrm{if}\;\;0\leq t\leq t_{e}\\ {\ddot{\varphi }}_{t} & \mathrm{if}\;\;t>t_{e} \end{array}\right. \end{array}$$

with ${\ddot{\varphi }}_{t}$ defined in Appendix A.1, vanishes. The expressions of the time ($t_{p}$) and the size (${\dot{C}}_{p}$) of the peak are available in Appendix A.2 for the general situation (ν≠0 and ρ≠0), as well as for limiting cases.

The number of detected cases $C_{t}$ is the basic data reported during an epidemic. Once this has been modeled, various epidemic related quantities can be inferred upon introduction of disease related parameters (e.g., detection of infectives, recoveries and deaths) and demographic parameters (e.g., natural mortality, births and immigration).

### 2.2. Infectives, Epidemic Latency Period and Active Cases

Since only a fraction of infectives are identified at a time *t*, the number $I_{t}$ of infective individuals in a target population is obtained using (6) as $I_{t}=\delta^{-1}{\dot{C}}_{t}$ [5], which reads

(8) $$\begin{array}{ccc} I_{t} & = & \left\{\begin{array}{cc} \;I_{0}e^{\omega_{0}t}\;\;\;\;\; & \mathrm{if}\;\;t\leq t_{e}\\ \delta^{-1}{\dot{\varphi }}_{t}\;\;\; & \mathrm{if}\;\;t>t_{e} \end{array}\right. \end{array}$$

where $I_{0}=\delta^{-1}\omega_{0}e^{-\omega_{0}\tau_{0}}$ is the number of infectives at the outbreak (t=0) and δ∈(0,1] is the detection rate assumed constant along the epidemic course (after the outbreak). Note that the number of infectives before the outbreak (t<0) is obtained by back extrapolation as $I_{t}=I_{0}e^{\omega_{0}t}$, i.e., considering an exponential growth before the outbreak [14].

We refer to the time from the appearance of the first infectious case in the population (“patient zero”) to the outbreak as the “epidemic latency period”. An estimate of the duration $t_{o}$ of this period is obtained by setting $I_{t}=1$ [14]. By (8), the duration of the epidemic latency period is estimated by $t_{o}=\omega_{0}^{-1}\log I_{0}$, which on using (4) simplifies to

(9) $$\begin{array}{c} t_{o}=\frac{\log{\dot{\varphi }}_{e}-\log\delta }{\omega_{0}}-t_{e}. \end{array}$$

The number of detected and active cases, i.e., individuals tested positive and in isolation at a hospital or at home at time *t*, is denoted $Q_{t}$ following Hethcote et al. [22] for “Quarantined” state, although we refer to $Q_{t}$ as “Actives”. Given the detected cases $C_{t}$ in (1), $Q_{t}$ satisfies

(10) $$\begin{array}{c} {\dot{Q}}_{t}={\dot{C}}_{t}-\left(\alpha_{t}+\epsilon_{t}\right)Q_{t} \end{array}$$

where $\alpha_{t}$ is the recovery rate and $\epsilon_{t}$ is the death rate (natural and disease-related mortality) of actives. Indeed, following Tovissodé et al. [5], we allow the removal rates $\alpha_{t}$ and $\epsilon_{t}$ from $Q_{t}$ to be time varying. This is appropriate when recovery and death data are available in addition to the reported positive cases per unit time. The two rates have here the logistic forms

(11) $$\begin{array}{ccc} \alpha_{t} & = & {\left[1+e^{-(\kappa_{0}+\kappa t)}\right]}^{-1} \end{array}$$

(12) $$\begin{array}{ccc} \epsilon_{t} & = & {\left[1+e^{-(\lambda_{0}+\lambda t)}\right]}^{-1}. \end{array}$$

The number of active cases is then given by (see Appendix B for details)

(13) $$\begin{array}{ccc} Q_{t} & = & \left\{\begin{array}{cc} \left[Q_{0}F_{0}+\omega_{0}\int_{0}^{t}e^{\omega_{0}\left(r-\tau_{0}\right)}F_{r}dr\right]F_{t}^{-1}\; & \;\;\;\;\;\mathrm{if}\;\;0<t\leq t_{e}\\ \left[Q_{e}F_{t_{e}}+\int_{t_{e}}^{t}{\dot{\varphi }}_{r}F_{r}dr\right]F_{t}^{-1}\; & \mathrm{if}\;\;t>t_{e} \end{array}\right. \end{array}$$

where $Q_{0}$ is available from Equation (A3) and represents the number of persistent cases from previous epidemic waves (isolated actives) at the outbreak of the target epidemic wave (e.g., $Q_{0}=0$ for a new disease-related epidemic) and $F_{t}$ is defined as

(14) $$\begin{array}{c} F_{t}=\left\{\begin{array}{cc} e^{(\alpha_{0}+\epsilon_{0})t}\; & \;\mathrm{if}\;\;\kappa =0\;\mathrm{and}\;\lambda =0\\ e^{\alpha_{0}t}{\left(1+e^{\lambda_{0}+\lambda t}\right)}^{1/\lambda }\; & \;\mathrm{if}\;\;\kappa =0\;\mathrm{and}\;\lambda \neq 0\\ {\left(1+e^{\kappa_{0}+\kappa t}\right)}^{1/\kappa }e^{\epsilon_{0}t}\; & \;\mathrm{if}\;\;\kappa \neq 0\;\mathrm{and}\;\lambda =0\\ {\left(1+e^{\kappa_{0}+\kappa t}\right)}^{1/\kappa }{\left(1+e^{\lambda_{0}+\lambda t}\right)}^{1/\lambda }\; & \;\mathrm{if}\;\;\kappa \neq 0\;\mathrm{and}\;\lambda \neq 0 \end{array}\right.. \end{array}$$

### 2.3. Overall Epidemic Dynamics

The dynamics of an epidemic, as expressed by the variations of the infectives $I_{t}$, is determined by the combination of the transmission rate (new infections) and the average residence time, i.e., the average duration from infection to isolation, recovery or death. The core parameter to summarize these dynamics is, at moment *t*, the reproduction number denoted $R_{t}$, which is indeed crucial for quantifying the intensity of control measures required to control an epidemic [7].

The reproduction number is defined as the average number of secondary cases generated by a primary case. With a view to derive $R_{t}$ under the growth model in (1), we first consider an overall picture of the target population in order to enlighten the sources (transmission and removal) of the variations of $I_{t}$ as given in (8).

#### 2.3.1. The SIQR Model

**Figure 1:**
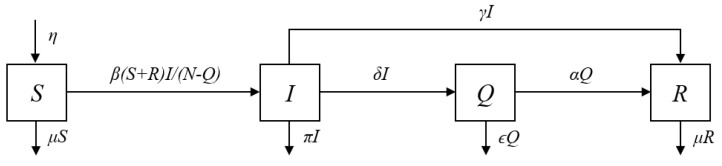
Transfer diagram for a SIQR model with quarantine-adjusted incidence. *S* is the class of susceptibles, *I* is the class of infectives, *Q* is the class of detected active cases, i.e., individuals tested positive and in isolation at a hospital or at home and *R* is the class of individuals who contracted the disease, were detected or not, and have recovered. The individuals in class *R* are considered permanently immune.

Following the authors of [5,14], we consider the Susceptible–Infectious–Quarantined–Recovered (SIQR) model of Hethcote et al. [22] to obtain a picture of the different states of individuals in a target population. We use the “quarantine-adjusted incidence” version [22] of this model since the underlying transmission mechanism explicitly recognizes the isolation of detected cases. In this framework, letting $N_{t}$ denote, at time *t*, the size of the target population (assumed finite but large), $N_{t}$ satisfies

(15) $$\begin{array}{c} N_{t}=S_{t}+I_{t}+Q_{t}+R_{t} \end{array}$$

where $S_{t}$ is the size of the class of susceptible individuals, $I_{t}$ is the class of infectives, $Q_{t}$ is the size of the class of detected active cases and $R_{t}$ is the size of the class of individuals who recovered (both detected and not detected). We assume that the infection has zero latent period (susceptible individuals become infectious as soon as they become infected). The individuals in the classes *R* are assumed permanently immune within the period of time considered. It is also assumed that known active cases (in the class *Q*) do not mix with other classes and do not infect the susceptibles (i.e., the transmission rate from *Q*-class individuals is considered negligible). The corresponding SIQR model is described by the following set of nonlinear differential equations [22]

(16) $$\begin{array}{ccc} {\dot{S}}_{t} & = & \eta -\beta_{t}\left(S_{t}+R_{t}\right)I_{t}/\left(N_{t}-Q_{t}\right)-\mu S_{t} \end{array}$$

(17) $$\begin{array}{ccc} {\dot{I}}_{t} & = & \left[\beta_{t}\left(S_{t}+R_{t}\right)/\left(N_{t}-Q_{t}\right)-\left(\gamma +\delta_{t}+\pi \right)\right]I_{t} \end{array}$$

(18) $$\begin{array}{ccc} {\dot{Q}}_{t} & = & \delta_{t}I_{t}-\left(\alpha_{t}+\epsilon_{t}\right)Q_{t} \end{array}$$

(19) $$\begin{array}{ccc} {\dot{R}}_{t} & = & \gamma I_{t}+\alpha_{t}Q_{t}-\mu R_{t} \end{array}$$

where η is the recruitment rate of susceptibles (births and immigration); $\beta_{t}$ is the total number of adequate contacts (i.e., contacts sufficient for transmission) per unit time; μ is the per capita natural mortality rate; $\alpha_{t}$ and γ are the recovery rates from actives $Q_{t}$ and infectives $I_{t}$ respectively; $\epsilon_{t}$ and π are the death rates (natural and disease-related) for actives $Q_{t}$ and infectives $I_{t}$ respectively; and $\delta_{t}$ is the detection rate which is null ($\delta_{t}=0$) for t<0 and equals $\delta_{t}=\delta$ for t≥0. Note that (18) is the same as (10) for t≥0. Unlike in [22], we allow the transmission rate $\beta_{t}$ to be time varying as a consequence of the form of the number of infectives $I_{t}$ already available in (8). The transfer diagram for this SIQR model is shown in Figure 1.

The system (16)–(19) always has the disease-free equilibrium $P_{0}=\left(S=\eta /\mu ,I=0,Q=0,R=0\right)$, i.e., in the absence of the disease, the population size $N_{t}$ approaches the carrying capacity $N^{*}=\eta /\mu$. Further discussion of the equilibria of the system are given in Appendix C.1. The availability of the number of infectives in Equation (8) makes it possible to solve the system (16)–(19). Indeed, from (17), the transmission rate, i.e., the number of adequate contacts per unit time (for $I_{t}>0$) is given by

(20) $$\begin{array}{c} \beta_{t}=\left(\gamma +\delta_{t}+\pi +\frac{{\dot{I}}_{t}}{I_{t}}\right)\left(1+\frac{I_{t}}{S_{t}+R_{t}}\right). \end{array}$$

From (20), and using the same approach considered to find the number $Q_{t}$ of active cases in Equation (13) from the number $I_{t}$ of infectives in Equation (8), the expressions of the number $S_{t}$ of susceptibles, the number $R_{t}$ of recovered individuals and the total number of persons infected during an epidemic wave can be obtained (see Appendix C.2 for details).

#### 2.3.2. The Effective Reproduction Number

From the definition of the effective reproduction number as the average number of secondary cases generated by a primary case, the threshold $R_{t}$ corresponds to the product of the transmission rate $\beta_{t}$ and the average residence time $1/(\gamma +\delta_{t}+\pi )$ in the class of infectives, i.e.,

$$\begin{array}{c} R_{t}=\beta_{t}/\left(\gamma +\delta_{t}+\pi \right). \end{array}$$

This effective reproduction number is sometimes referred to as a “quarantine” reproduction number [22] or simply a “control” reproduction number to acknowledge the influence of isolation of identified infectives, and other control measures, if any [15]. The basic reproduction number defined as the average number of secondary infections produced when one primary infectious individual enters a completely susceptible population ($S_{o}=N_{o}-1$, $I_{o}=1$, $Q_{o}=0$, $R_{o}=0$), is here given by $R_{o}=\left(1+\frac{\omega_{0}}{\gamma +\pi }\right)N_{o}/\left(N_{o}-1\right)$. This expression is simplified, assuming $N_{o}/\left(N_{o}-1\right)=1$ for the sake of beauty [23] and mostly because $N_{o}$ is large (recall this is a model assumption), as

(21) $$\begin{array}{c} R_{o}=1+\frac{\omega_{0}}{\gamma +\pi }. \end{array}$$

During the epidemic latency period ($t_{o}<t<0$) where the growth is exponential (${\dot{I}}_{t}/I_{t}=\omega_{0}$) and the detection rate is $\delta_{t}=0$, the time-varying reproduction number is given by

(22) $$\begin{array}{c} R_{t}=\left(1+\frac{\omega_{0}}{\gamma +\pi }\right)\left(1+\frac{I_{t}}{S_{t}+R_{t}}\right)\;\;\mathrm{for}\;\;-t_{o}\leq t<0. \end{array}$$

From the outbreak, the time-varying effective reproduction number during the remaining of the exponential phase has the same form

(23) $$\begin{array}{c} R_{t}=\left(1+\frac{\omega_{0}}{\gamma +\delta +\pi }\right)\left(1+\frac{I_{t}}{S_{t}+R_{t}}\right)\;\;\mathrm{for}\;\;0\leq t\leq t_{e}. \end{array}$$

It appears from (22) and (23) that $R_{t}>1$ during the whole exponential growth phase as expected. During the sub-exponential growth phase, the time-varying effective reproduction number is given by

(24) $$\begin{array}{c} R_{t}=\left(1+\frac{z_{t}}{\gamma +\delta +\pi }\right)\left(1+\frac{I_{t}}{S_{t}+R_{t}}\right)\;\;\mathrm{for}\;\;t>t_{e} \end{array}$$

where $z_{t}={\ddot{\varphi }}_{t}/{\dot{\varphi }}_{t}$ (see Appendix A.1).

#### 2.3.3. Epidemic Peak

The peak of new infections occurs when the second derivative of the total number of infected persons (since the beginning of the epidemic) vanishes. This peak time denoted $t_{new}$ satisfies $t_{new}>t_{e}$ and is the solution of (see details in Appendix C.3)

(25) $$\begin{array}{c} \left(\gamma +\delta +\pi \right){\ddot{\varphi }}_{t}+{\dddot{\varphi }}_{t}=0 \end{array}$$

which can be solved for *t* using a numerical root finding routine such as the R [24] function *uniroot* or the Matlab [25] function *fzero*. Afterwards, the peak size ${\dot{T}}_{new}$ (the maximum number of new infections per unit time) is obtained by inserting $t_{new}$ in (A14).

### 2.4. Long-Term Epidemic Dynamics

The specification of the growth model in (1) to an epidemic implicitly assumes that the number of infectives in (8) peaks at time $t_{p}$ and then approaches zero. The decay of the infectives after the peak can happen at various rates, depending on the growth pattern (determined by contacts between the infectives and the susceptibles or intermediate hosts), the response of the infected individual’s organism (natural or induced with medicine or a vaccine) to the disease (recovery and death process) and the testing efforts (detection followed by isolation). There are actually two alternative paths from a disease-related state (i.e., $I_{t}>0$) toward the unique (disease-free) equilibrium $P_{0}$: transmissions either stop ($R_{t}$ reaches zero) or continue fro a long time at a rate which cannot sustain an epidemic ($0<R_{t}\leq 1$). These two scenarios are discussed further in Appendix C.4.

### 2.5. Statistical Model and Inference

To allow likelihood inference in the growth models in (1) using observed epidemiological data, we follow Tovissodé et al. [5] and assign to new reported cases $Y_{t}$ (t=1,2,⋯,n) a log-normal distribution with probability density function (pdf)

(26) $$\begin{array}{c} f_{Y}\left(Y_{t}|\theta \right)=\frac{1}{\sigma \left(Y_{t}+1\right)\sqrt{2\pi }}\exp\left(-\frac{1}{2}{\left[\frac{\log\left(Y_{t}+1\right)-\log\left({\dot{C}}_{t}+1\right)}{\sigma }+\frac{\sigma }{2}\right]}^{2}\right) \end{array}$$

where σ>0 is a dispersion parameter (standard deviation at logarithmic scale). This specification yields the mean $E\left[Y_{t}\right]={\dot{C}}_{t}$ and the variance $Var\left[Y_{t}\right]={\left({\dot{C}}_{t}+1\right)}^{2}\left(e^{\sigma^{2}}-1\right)$ while allowing null values of $Y_{t}$. In addition, the numbers of new recoveries $G_{t}$ and new deaths $M_{t}$ from known active cases $Q_{t}$ (t=1,2,⋯,n) are modeled using logistic regression models with probability mass functions (pmf)

(27) fG(Gt|θ,Qt−1,Yt)=Qt−1+YtGtαtGt(1−αt)Qt−1+Yt−Gt

(28) fM(Mt|θ,Qt−1,Yt)=Qt−1+YtMtϵtMt(1−ϵt)Qt−1+Yt−Mt

where $\alpha_{t}={\left[1+e^{\kappa_{0}+\kappa t}\right]}^{-1}$ and $\epsilon_{t}={\left[1+e^{\lambda_{0}+\lambda t}\right]}^{-1}$. The parameter vector indexing the pdf in (26) and the conditional pmf in (27) and (28) is $\theta ={\left(\Omega ,\omega ,\nu ,\rho ,\tau ,t_{e},\sigma ,\kappa_{0},\kappa ,\lambda_{0},\lambda \right)}^{⊤}$ when the generic growth curve is considered for the sub-exponential growth phase. If a special case of the generic growth curve is desired, the corresponding restricted parameters must be withdrawn from θ. For instance, the use of a hyper-logistic growth curve (ν=1) implies $\theta ={\left(\Omega ,\omega ,\rho ,\tau ,t_{e},\sigma ,\kappa_{0},\kappa ,\lambda_{0},\lambda \right)}^{⊤}$. Given $Q_{0}$, the conditional log-likelihood of an observed series $\left\{Y_{t},G_{t},M_{t}\right\}$ with t=1,2,⋯,n, as a function of the parameter θ is

(29) $$\begin{array}{ccc} \ell (\theta ) & = & \ell_{Y}\left(\theta \right)+\ell_{G}\left(\theta \right)+\ell_{M}\left(\theta \right) \end{array}$$

(30) $$\begin{array}{ccc} \mathrm{where}\;\ell_{Y}\left(\theta \right) & = & \sum_{t=1}^{n}\log f_{Y}\left(Y_{t}|\theta \right) \end{array}$$

(31) $$\begin{array}{ccc} \ell_{G}\left(\theta \right) & = & \sum_{t=1}^{n}\log f_{G}\left(G_{t}|\theta ,Q_{t-1},Y_{t}\right) \end{array}$$

(32) $$\begin{array}{ccc} \ell_{M}\left(\theta \right) & = & \sum_{t=1}^{n}\log f_{M}\left(M_{t}|\theta ,Q_{t-1},Y_{t}\right). \end{array}$$

The conditional maximum likelihood estimate $\hat{\theta }$ of θ can be obtained using an optimization algorithm to maximize the log-likelihood function *ℓ*. Note that the three components of ℓ(θ) are separable and can be maximized independently. In other words, the parameter vector θ has the partition $\theta ={\left(\theta_{Y}^{⊤},\theta_{G}^{⊤},\theta_{M}^{⊤}\right)}^{⊤}$ and the maximum likelihood estimates of the components $\theta_{Y}={\left(\Omega ,\omega ,\nu ,\rho ,\tau_{0},t_{e},\sigma \right)}^{⊤}$, $\theta_{G}={\left(\kappa_{0},\kappa \right)}^{⊤}$ and $\theta_{M}={\left(\lambda_{0},\lambda \right)}^{⊤}$ can be obtained by maximizing $\ell_{Y}$, $\ell_{G}$ and $\ell_{M}$ respectively.

Since both the binomial and the log-normal distributions belong to the exponential family, we consider the common deviance statistic used in Generalized Linear Models [26] for checking the goodness-of-fit of the log-normal model associated to $Y_{t}$ and the binomial models associated to $G_{t}$ and $M_{t}$. For the selection of the parsimonious model agreeing with the observed data, we consider the likelihood ratio statistic [27]. Further details on the deviance statistic and the likelihood ratio test are given in Appendix D.

## 3. Application to COVID-19 Data of Western Africa

### 3.1. Context and Objectives

The Western African region has 16 countries (Benin, Burkina-Faso, Cape Verde, Côte d’Ivoire, Gambia, Ghana, Guinea, Guinea-Bissau, Liberia, Mali, Mauritania, Niger, Nigeria, Senegal, Sierra Leone and Togo), covering 6,140,178 km^2^ with a population of about 402,555,230 inhabitants [28] (Table 1).

The first COVID-19 patient was formally identified in Western Africa in late (27) February 2020. We considered COVID-19 daily infection (PCR-confirmed cases on the day of reporting), recovery and death data, from 28 February to 31 August 2020, obtained from the Global Rise of Education Platform [29]. This period roughly corresponds to the first wave of the COVID-19 pandemic in the region [30]. We concentrated on these six months of data since the proposed modeling framework has been designed for a single epidemic wave. As of 31 August 2020, the region had 167,684 confirmed cases, among which 83.64% recovered and 1.52% died (Table 1). Although the region is heterogeneous, we treated it as if it were homogeneous. Indeed, it must be kept in mind that the reported COVID-19 cases occurred in small clusters concentrated in the main cities of each country. Hence, the sparsity of the data for the whole region actually reflect data sparsity at national and city levels.

The purpose of this analysis is to demonstrate, by example, the use of the proposed modeling framework. The specific aims are: (i) to model COVID-19 case reporting data (daily PCR-confirmed positives, recoveries and deaths) from Western Africa (28 February to 31 August 2020); and (ii) to evaluate the transmission pattern of the disease. Most West African governments have planned and subsequently implemented several control measures, either before or overlapping with the time of diagnosis of the first national cases [31]. The main sequence of public health and movement restriction measures taken by West African governments during the considered period includes personal hygiene and social distancing recommendations and isolation/lockdown (Table 2). The adoption of these containment measures followed a sustained increment during late March 2020. The modeling results are used to discuss the effectiveness of the containment measures and the implications for the control of the further spread of COVID-19 in West African countries.

### 3.2. Data Analysis

All computations and statistical analyses were performed in R software [24]. The significance level of statistical tests was set to 5%.

#### 3.2.1. Model Fitting

We fitted the generic growth curve to the daily new infections $Y_{t}$. We used the *optim* routine of R software to maximize the log-likelihood (30). We also fitted three of its special cases (Bertalanffy–Richards, hyper-logistic and hyper-Gompertz), which were compared to the generic model fit using likelihood ratio tests. Instead of directly maximizing the log-likelihoods (31) for ${\hat{\theta }}_{G}$ and (32) for ${\hat{\theta }}_{M}$ with the *optim* routine, we used the *glm* routine of R with the family specification “family = binomial(logit)”. Since COVID-19 was a new disease in 2020, we considered the number of known active cases $Q_{0}=0$ at t=0 in (27) and (28). We plotted the daily new positives, recoveries, deaths and actives to provide graphical insights in the fitted models.

#### 3.2.2. Overall Epidemic Dynamics

We analyzed the overall dynamics of the COVID-19 epidemic in West Africa using the mechanistic SIQR model described in Section 2.3. The rate parameters δ (detection rate), γ and π (recovery and death rates in infected but non-detected individuals) cannot be estimated using only the available data sequence $\left\{Y_{t},G_{t},M_{t}\right\}$ (daily new positives, recoveries and deaths) without additional assumptions on their relationships with the rate parameters for detected cases ($\alpha_{t}$ and $\epsilon_{t}$). We obtained from the literature δ=0.009 [30] and γ+π=1/10 [14,30] and assumed that the ratio of the daily recovery probability to the daily death probability in non detected infectives is equal to this ratio in the detected individuals at outbreak, i.e., before the implementation of treatments, if any. From $\gamma /\pi =\alpha_{0}/\epsilon_{0}\approx 5.1495$, we obtained γ=1/11.9419 and π=1/61.4953.

Two demographic parameters are required in the SIQR model: the daily recruitment rate of susceptibles (through births and immigration) η (individuals/day) and the per capita natural mortality rate μ (day^−1^). Using the birth rate $\rho_{b}$ (total births and net immigrations in a period of length *L* divided by the average population size $\bar{N}$ during this period), the recruitment rate η was estimated by

(33) $$\begin{array}{c} \eta =\frac{r_{b}\bar{N}}{L}. \end{array}$$

Under “natural” (i.e., disease-free) conditions where $N_{t}=S_{t}$, the variation ΔN of the population size $N_{t}$ over a period of length *L* satisfies

(34) $$\begin{array}{ccc} \Delta N & = & \left(\frac{\eta }{\mu }-N_{i}\right)\left(1-e^{-\mu L}\right) \end{array}$$

where $N_{i}$ is the population of West Africa at the beginning of the period. The Equation (34) follows by (A5) with $I_{0}=0$. The variation ΔN of the population size is given by $\Delta N=r_{b}\bar{N}-r_{d}\bar{N}$, where $r_{b}\bar{N}$ represents the total recruitment during the period and $r_{d}\bar{N}$ represents the total number of deaths with $\rho_{d}$ the mortality rate (individuals/day). Consequently, μ can be obtained by solving (34) for μ using $\Delta N=\left(r_{b}-r_{d}\right)\bar{N}$.

We considered L=365.25 days, $\bar{N}=401,861,254$, $N_{i}=397,429,929$ [28]. Using the annual birth (32.816/1000) and death rates (7.952/1000) [32] and the net annual immigrations (−177,000 individuals) in West Africa [28], we obtained the rates $r_{b}=\left(32.816/1000\right)-\left(177,000/\bar{N}\right)=32.371/1000$ and $r_{d}=7.952/1000$. By (33) and (34), we then found and used for our analyses on West Africa, η=35,615.35 individuals/day and $\mu =2.1745\times 10^{-5}$ day^−1^. We plotted the daily number of new infections, infectives and recovered individuals, as well as the reproduction number in the West African population.

#### 3.2.3. Standard Error and Confidence Interval

Standard errors were obtained for quantities calculated using estimated model parameters by the delta method [33]. For a positive definite parameter or calculated quantity ϕ in general, we first found the estimate $\hat{\phi }$ and its logarithmic scale-standard error ${\hat{\sigma }}_{\phi }$ by the delta method and computed its logarithmic scale-mean given by ${\hat{\mu }}_{\phi }=\log\hat{\phi }-0.5{\hat{\sigma }}_{\phi }^{2}$. We then obtained the bounds of its shortest confidence interval as described by Dahiya and Guttman [34].

### 3.3. Results

#### 3.3.1. Growth Curve for New Positives and Logistic Regressions for Removals

The results of the likelihood ratio tests comparing the generic growth model (1) against its closest special cases are presented in Table 3. The growth model involving the generic growth curve was retained. Indeed, the combination of an early exponential growth and the generic growth models was found to be the best growth model for the new positive cases in West Africa, as compared to the combinations of the exponential growth with the Bertalanffy–Richards, hyper-logistic and hyper-Gompertz growth models (Table 3; *p*-value < 0.001).

The deviance based $\chi^{2}$ test for overall goodness-of-fit (Table 4) indicates a lack-of-fit (*p*-value < 0.001), with an overall adjusted-deviance reduction ratio of $r_{dev}^{2}=11.60\%$. Looking for the sub-models, we noticed that the estimated growth curve is significantly different from the corresponding null model fit (*p*-value < 0.001) and does not lack fit (*p*-value = 0.6115). Indeed, the adjusted-deviance reduction ratio is $r_{dev}^{2}=95.26\%$ (the adjusted-coefficient of determination is $r_{a}^{2}=99.96\%$). The overall lack of fit is due to the logistic regression fits for the daily recoveries ($r_{dev}^{2}=9.25\%$) and deaths ($r_{dev}^{2}=49.08\%$). We nevertheless kept these fits because there are significantly different from the corresponding null model fits (*p*-value < 0.001).

The maximum likelihood estimates of the generic growth model and logistic regression model parameters are presented in Table 5. The Wald test results (Table 5) agree with the likelihood ratio tests considered to select the growth model for the sub-exponential growth phase. Indeed, the 95% confidence bounds for the parameters ν (CI(ν)=[2.77,4.82]) and ρ (CI(ρ)=[0.09,0.15]) indicate that none of the Bertalanffy–Richards growth model (ρ→0), the hyper-logistic growth model (ν=1), the logistic growth model (ρ→0, ν=1), the hyper-Gompertz growth model (ν→0, $\omega \nu^{1+\rho }\to \tilde{\omega }$) and the Gompertz growth model (ρ→0, ν→0, $\omega \nu \to \tilde{\omega }$) are appropriate for this dataset.

**Figure 2:**
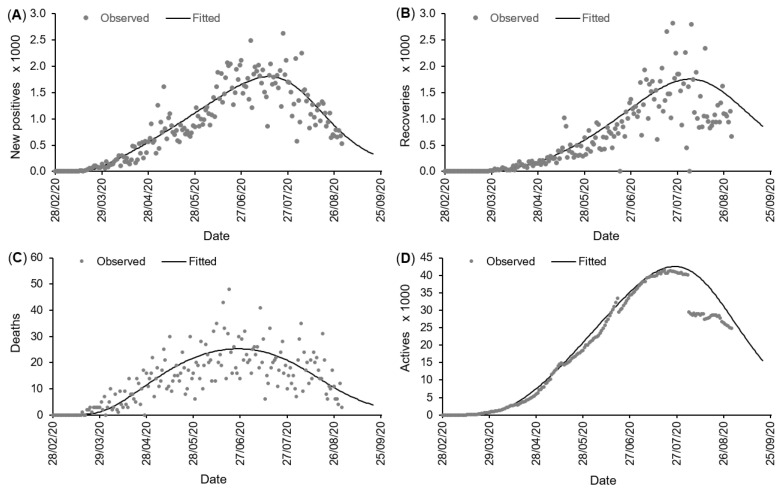
Records of new positive cases ${\dot{C}}_{t}$ (**A**), daily recoveries $\alpha_{t}Q_{t}$, (**B**), daily deaths $\epsilon_{t}Q_{t}$ (**C**) and known actives cases (quarantined at home/hospital) $Q_{t}$ (**D**) in COVID-19 daily case reporting data from West Africa (28 February to 31 August 2020). The fitted curves are based on a combination of an early exponential growth model and a generic growth model with log-normal error structure for the daily new positive cases ${\dot{C}}_{t}$, two logistic regression models for the probabilities of recovery ($\alpha_{t}$) and death ($\epsilon_{t}$) and the combination of ${\dot{C}}_{t}$, $\alpha_{t}$ and $\epsilon_{t}$ (using (13)) for actives $Q_{t}$. Two outlying data points (6006 recoveries on 20 June 2020 and 11,468 recoveries on 4 August 2020) were removed from the graph (**B**) for a better visualization.

The exponential growth phase lasted about one month (${\hat{t}}_{e}=29.48$, $CI\left(t_{e}\right)=\left[26.94,31.79\right]$ days) after the outbreak (Table 5). The growth curve fitted to the cumulative positive cases is given by

(35) $$\begin{array}{c} C_{t}\;=\;\left\{\begin{array}{c} \;e^{0.1660\times (t+7.2208)}\;\;\;\;\;\;\;\mathrm{if}\;0<t\leq 29.48\\ 200.3128+\frac{191,290.8}{{\left\{1+{\left[1+0.0067\times (t-171.3210)\right]}^{8.3185}\right\}}^{0.2656}}\;\;\mathrm{if}\;t>29.48 \end{array}\right. \end{array}$$

where *t* is the time (day) from the outbreak. Figure 2A shows the daily confirmed positive cases and the fitted growth curve based on a log-normal error structure. The observed peak of new positives happened 148 days after the outbreak (24 July 2020) and amounted to 2626 positive cases. However, the number of positive cases showed a high variability around this date (16–29/07/2020), with most daily records roughly ranging between 1600 and 2000 new positive cases (Figure 2A) around an average of 1803 cases (with standard error SE=86.48). The estimated peak time for the new positive cases was around 15 July 2020, i.e., about 139 days after the outbreak (Table 6), and the estimate of the peak size is about 1805 new positive cases ($CI\left({\dot{C}}_{p}\right)=\left[1643.19,1969.86\right]$). Assuming a log-normal distribution, the 95% prediction interval for the peak size is $PI\left({\dot{C}}_{p}\right)=\left[1368.93,2669.55\right]$ new positive cases, which includes the observed value. The 95% prediction interval for the peak time is $PI\left(t_{p}\right)=\left[126.59,151.65\right]$ days, which also includes the observed peak time.

**Figure 3:**
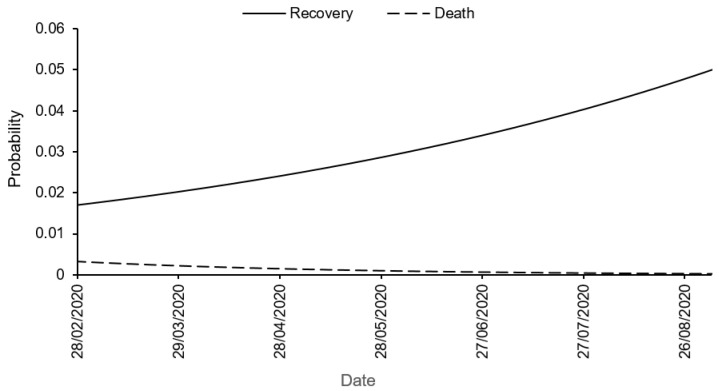
Fitted probabilities of recovery and death in COVID-19 daily case reporting data from West Africa (28 February to 31 August 2020). The fits are based on two logistic regression models.

Based on the logistic regression parameters shown in Table 5, the probabilities of removals from the actives (quarantined) are shown in Figure 3. The probabilities of recovery and death are ${\hat{\alpha }}_{0}=0.0169$ and ${\hat{\epsilon }}_{0}=0.0033$, respectively, at outbreak (t=0). The recovery probability then improved, with an odd ratio (recover/not recover) increasing on average by 0.59% (CI(κ)=[0.58,0.61]%) each day. The death probability on the contrary decreased, with an odd ratio (die/not die) decreasing on average by 1.26% (CI(λ)=[−1.37,−1.15]%) each day.

Figure 2B,C shows the removals (daily recovery and death) and the fitted values based on the logistic regression models for removal probabilities. We noticed that the lack-of-fit (indicated by the residual deviance test) is due to the very large variability of the observed daily proportions of recoveries and deaths. However, despite the lack-of-fit in the logistic regression fits, the use of the related recovery and death probabilities ($\alpha_{t}$ and $\epsilon_{t}$) along with the fitted growth curve (${\dot{C}}_{t}$), resulted in fitted active cases ($Q_{t}$) agreeing to a large extent with the observed daily actives (Figure 2D), with an adjusted-coefficient of determination of 97.08%. The peak of known active cases ($Q_{t}$) was on 19 July 2020 and amounted to 41,435 actives. The fitted peak is about 42,507 actives around 26 July 2020 (Table 6). The 95% prediction interval is $PI\left(Q_{max}\right)=\left[34,807.25,50,893.54\right]$ actives for the maximum of active cases and $PI\left(t_{Q_{max}}\right)=\left[139.92,159.71\right]$ days for the peak time $t_{Q_{max}}$ (16 July to 5 August 2020).

#### 3.3.2. Overall Epidemic Dynamics

The estimate of the duration of the epidemic latency period (delay between the arrival of the first infectious individual and outbreak) is about 25 days ($CI\left(t_{o}\right)=\left[19.91,29.87\right]$ days; see Table 6). Accordingly, the first imported COVID-19 case(s) in West Africa likely entered the region during the last week of January and the first week of February (28 January–7 February) 2020. The estimate of the basic reproduction number is ${\hat{R}}_{o}=2.66$ ($CI\left(R_{o}\right)=\left[2.60,2.69\right]$). At outbreak, the number of infectives in the region is estimated at about 61 ($CI\left(I_{0}\right)=\left[47.98,75.05\right]$) infectives. The estimate of the control reproduction number during the exponential growth phase after the outbreak is ${\hat{R}}_{0}=2.52$ ($CI\left(R_{0}\right)=\left[2.29,2.76\right]$).

**Figure 4:**
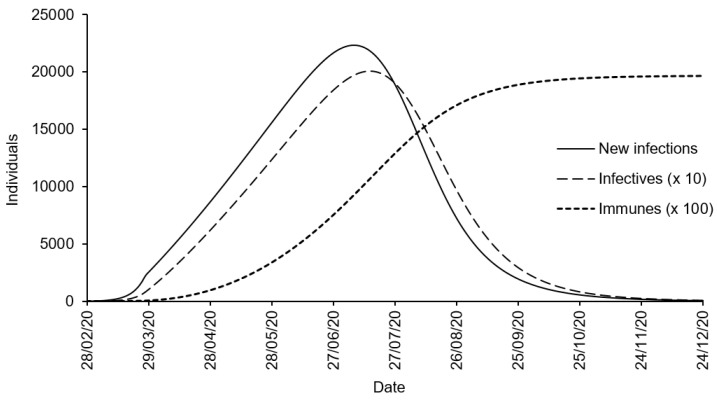
Estimates of the daily number of new infections, infectives and recovered individuals using the COVID-19 daily case reporting data from West Africa (28 February to 31 August 2020). The estimates are based on a SIQR model (see (16)–(19)) with rate parameters δ=0.009 day^−1^ (detection rate), γ=1/11.9419 day^−1^ (recovery rate for non detected), π=1/61.4953 day^−1^ (death rate for non detected), η=35,615.35 individuals/day (recruitment rate) and $\mu =2.1745\times 10^{-5}$ day^−1^ (natural mortality rate).

Figure 4 shows the curves of the daily number of new infections (${\dot{T}}_{t}$), the daily number of infectives ($I_{t}$) and the immune fraction of the population ($R_{t}=K_{t}+U_{t}$). As expected, the peak in new infections occurred before the peak in detected infected individuals (observed 143 days after the outbreak). Indeed, the number of new infections peaked about 131 days after the outbreak ($CI\left(t_{new}\right)=\left[126.18,136.11\right]$ days), i.e., around 7 (2–12) July 2020, to about 22,353 ($CI\left({\dot{T}}_{max}\right)=\left[20,284.04,24,464.98\right]$) new infections. As of 31 August 2020, the number of known recoveries in the West African region was 140,249. The number of both known and unknown recovered people at this date is estimated at about 1,754,699 individuals ($CI\left(R_{186}\right)=\left[1,675,407.60,1,834,783.00\right]$), i.e., about 0.44% of the population in the region.

**Figure 5:**
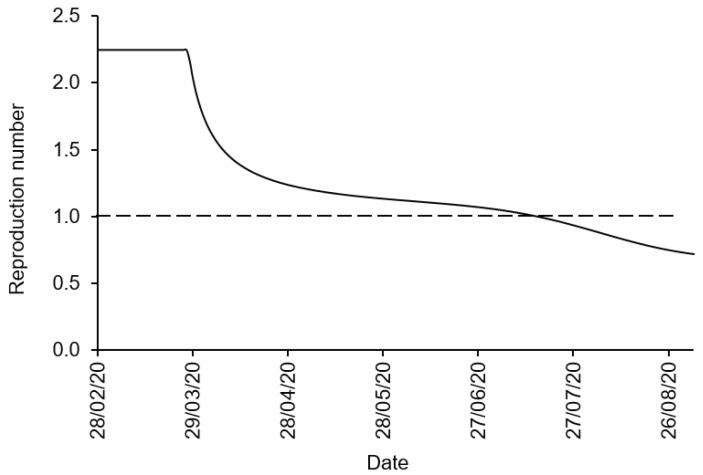
Time varying effective reproduction number of the 2020 COVID-19 epidemic in West Africa using daily case reporting data (28 February to 31 August 2020). The estimate is based on a SIQR model (see (16)–(19)) with rate parameters δ=0.009 day^−1^ (detection rate), γ=1/11.9419 day^−1^ (recovery rate for non detected), π=1/61.4953 day^−1^ (death rate for non detected), η=35,615.35 individuals/day (recruitment rate) and $\mu =2.1745\times 10^{-5}$ day^−1^ (natural mortality rate).

The time-varying effective reproduction number is shown in Figure 5. It appears that the effective reproduction number first decreased during the sub-exponential growth phase (from 2.52 on 27 February 2020), reaching 1 on 15 July and 0.66 on 31 August 2020. The effective reproduction number attained a minimum value of 0.61 on 29 September 2020 and then increased with a dynamics indicating $R_{\infty }=1$.

## 4. Discussion

The importance of mathematical models in understanding and predicting the course of an epidemic outbreak and in assessing the impacts of public health control measures has been well documented in the current context of the COVID-19 pandemic [15,35,36,37]. Whereas phenomenological modeling is limited in the scope of inference, compartmental modeling faces identifiability issues and is usually computationally intensive [38]. This study proposes a hybrid modeling framework which combines phenomenological and mechanistic modeling approaches to assess the dynamics of epidemic outbreaks while circumventing some of the limitations of each approach. We illustrate our description of the different epidemiological aspects that the hybrid modeling framework deals with using COVID-19 data from West Africa (28 February to 31 August 2020). It is worth noting that the heterogeneity of the West African region in terms of testing and reporting policies, especially for the first epidemic wave, is an important limitation for this application. This is systematically true for any regional assessment of the pandemic [15]. Our analysis aims to provide an overall view of the dynamics of the pandemic in the West Africa. However, the analysis of the data from each country may be conducted to obtain finer country-specific results (for some countries, these may significantly deviate from the overall trend).

The proposed modeling framework uses a combination of the exponential growth model for the initial dynamics of the epidemic and a generic growth curve [8] to capture the observed patterns in the number of detected positive individuals. This phenomenological model is flexible, includes many special cases and thus allows selecting the effective parsimonious model fitting the observed data based on likelihood ratio tests [27,39] or information criteria such as the Akaike’s Information Criterion [40]. The effectiveness of this approach to phenomenological modeling has been demonstrated on COVID-19 data [5]. Our application on COVID-19 data from West Africa nevertheless showed that the logistic regression of recoveries and deaths in the identified positive individuals against time can lack fit, as measured by an asymptotic $\chi^{2}$ test on the residual deviance statistic. Nevertheless, these fits can be improved by adding explanatories (different from time, but related to available health facilities) in the logistic regression models. The deterministic SIQR model [22] considered for mechanistic modeling explicitly acknowledges the isolation of the detected positive individuals. It does not, however, include an exposed (E) state as in the SEIQR model [41]. The use of the SEIQR model may provide better insights on the effectiveness of control measures since most of the measures first impact the exposition of susceptible individuals. In general, the proposed modeling approach can be extended by considering more complex models such as the SEIQR and the SIDARTHE model [42] instead of the SIQR model considered herein.

Among interest quantities provided by the hybrid modeling framework, we have the epidemic latency period $t_{o}$ (the time from the appearance of the first infectious case in the population to the outbreak). For the West African region, the result indicates that the first imported COVID-19 case(s) in West Africa likely entered the region around 28 January–7 February 2020. To the best of our knowledge, this is the first estimate of this duration in the region. This epidemic latency period is much lower than the 40 days estimated for Italy [14]. This is in line with the relatively late arrival of the virus in the region, compared to the Asian and European continents, and the prevention and detection measures anticipated by many West African governments [31]. We obtained a basic reproduction number ($CI\left(R_{o}\right)=\left[2.60,2.69\right]$) higher than the estimate ($CI\left(R_{o}\right)=\left[1.84,1.87\right]$) obtained by [15]. Our estimate is, however, closer to country-specific estimates obtained for Nigeria ($CI\left(R_{o}\right)=\left[2.37,2.47\right]$) [43] and Ghana ($CI\left(R_{o}\right)=\left[1.99,3.37\right]$) [44].

During the early phase of the epidemic after the outbreak in West Africa, the detection and isolation of a fraction of infected individuals reduced the reproduction number from $R_{o}$ to a control reproduction number of ${\hat{R}}_{0}=2.52$, i.e., about 5.26% decrease. We estimated the duration of this phase characterized by an exponential growth to be about one month after the outbreak. This implies that the control measures implemented by West African governments to limit the transmission of the disease were not effective on average before April 2020. Indeed, apart from measures taken to limit the importation of new positive individuals (travel bans), many actions to limit the local propagation of the disease were first implemented in late March 2020 [31] (e.g., curfew set up on 21 March in Burkina-Faso, on 23 March in Côte d’Ivoire, Mauritius and Senegal and on 26 March in Mali; city lockdown on 22 March in Ghana and on 29 March in Nigeria; isolation of the capital from the rest of the country in Côte d’Ivoire on 25 March 2020; and *cordon sanitaire* set up to isolate the south from the rest of the country on 30 March 2020 in Benin). Our results indicate that these measures started to impact the dynamics of the epidemic from early April 2020. However, the measures may have affected the transmission dynamics earlier, since the measures mainly limited the exposition of susceptible individuals to the disease.

After the exponential growth phase, the sub-exponential growth pattern allowed the epidemic to peak. The estimated peak time for the detected positive cases was around 15 July 2020, and close to the observed peak time (24 July 2020). This estimated date has a delay of about eight days with respect to the estimated peak time of new infections ($CI\left(t_{new}\right)=\left[126.18,136.11\right]$ days). This estimate is higher than the estimate ($CI\left(t_{new}\right)=\left[108,112\right]$ days) obtained by [30]. These contrasting results may be related to the more realistic SIQR model considered in this work as compared to the simpler SIR model used by Honfo et al. [30] who ignored the quarantine-adjustment of the disease incidence [22]. On the contrary, the estimated maximum number of new infections ($CI\left({\dot{T}}_{max}\right)=\left[20,284.04,24,464.98\right]$) agrees with the estimate ($CI\left({\dot{T}}_{max}\right)=\left[24,239,26,294\right]$ new infections) obtained by Honfo et al. [30].

Our results show that the time-varying effective reproduction number has decayed over April–August 2020, reaching 1 on about 15 July 2020 and 0.66 at the end of the considered period (31 August 2020). Based on the modeled dynamics, the effective reproduction number likely reached its minimum value 0.61 around 29 September 2020. However, the reproduction number likely increased again to approach $R_{\infty }=1$ in the long run. Overall, the various measures decided and enforced by different West African governments, against the first COVID-19 epidemic wave in the region, were able to contain the propagation of the disease (importation of new cases and local transmission) in five months.

However, the COVID-19 pandemic will remain an important issue for a long time, and local region’s endemic to the pathogen will likely appear in the long run. This is so because of the following factors: the re-opening of borders and airports in the region to limit the related economic feedback [45,46]; the relaxation of measures such as the ban of sport, political, cultural and religious gatherings [31,47]; and the natural evolution of the SARS-Cov-2 virus [48,49,50,51]. The limited resources and capacity of Sub-Saharan Africa countries in general [52,53,54] to immunize their population through vaccination will compound this threat in the region.

## 5. Conclusions

There are two common approaches to epidemiological modeling: phenomenological models and mechanistic models. This study proposes a hybrid framework which combines the two approaches, starting from fitting curves to observed data (confirmed positive cases, recoveries and deaths) and then providing an overall view of the epidemic dynamics by integrating the fitted curves into a compartmental model. The proposed approach allows estimating the delay between the appearance of the first infectious case in the population and the outbreak (“epidemic latency period”); the duration of period during which the epidemic growths exponentially; the basic and control reproduction numbers; and the peaks (time and size) in positive cases, active cases and new infections. An application to COVID-19 data from West Africa indicates that the hybrid modeling framework can be used to match effective control measures dictated by health policies with changes in the transmission dynamics of the studied disease.

## Figures and Tables

**Table 1:** Population size [28] and cumulative PCR-confirmed COVID-19 cases, deaths and recoveries in West Africa (28 February to 31 August 2020) [29].

Country	Population Size	Total Confirmed	Recoveries	Deaths
Nigeria	206,522,290	54,008	41,638	1013
Ghana	31,072,945	44,298	42,963	276
Côte d’Ivoire	26,428,999	18,067	16,699	117
Niger	24,269,389	1176	1088	69
Burkina-Faso	20,946,992	1368	1058	55
Mali	20,294,900	2776	2169	126
Senegal	16,776,618	13,611	9439	284
Guinea	13,160,021	9409	8447	59
Benin	12,123,200	2145	1738	40
Togo	8,293,924	1400	1005	28
Sierra Leone	7,989,949	2022	1594	70
Liberia	5,066,990	1304	872	82
Mauritania	4,659,052	7048	6464	159
Gambia	2,421,823	2963	1032	96
Guinea-Bissau	1,971,640	2205	1127	34
Cape Verde	556,498	3884	2916	40
West Africa	402,555,230	167,684	140,249	2548

**Table 2:** Main sequence of public health and movement restriction measures taken by West African governments during the first phase of the COVID-19 pandemic (until 31 August 2020).

Main Interventions	First Introduction (Country)	Implementation by the Last Country
State of health emergency and social distancing	22 March 2020 (Ghana)	30 March 2020 (Sierra Leone)
Setting up test sites and measures to quarantine suspected cases and isolate positive cases	25 February 2020 (Nigeria)	Early March 2020
Partial lockdown	18 March 2020 (Benin)	Late March
Curfew	20 March 2020 (Burkina Faso)	Not all countries
Reduced mobility and prohibition of social gatherings	15 March 2020 (Ghana)	Late March 2020
Land borders closure	20 March 2020 (Côte d’Ivoire)	30 March 2020 (Sierra Leone)
Wearing face mask in public mandatory	8 April 2020 (Benin)	14 May 2020 (Mauritania)
Systematic testing of target groups	22 March 2020 (Benin)	Late March to early April

Sources: https://hsfnotes.com/africa/2020/05/22/covid-19-initial-responses-of-certain-african-countries#page=1 (accessed on 4 April 2021) and [31].

**Table 3:** Likelihood ratio test results comparing the generic growth model [8] to three of its special cases.

Special Growth Model	Restriction	LRS	DF	*p*-Value
Bertalanffy–Richards	ρ→0	60.06	1	<0.001
Hyper-logistic	ν=1	240.33	1	<0.001
Hyper-Gompertz	ν→0, $\nu \omega^{\left(1+\rho \right)}\to \tilde{\omega }$	512.91	1	<0.001

Table notes: LRS, likelihood ratio statistic; DF, Degrees of freedom; $\tilde{\omega }$ stands for a positive constant; see Equation (1) for details on the parameters ν, ρ and ω.

**Table 4:** Deviance based goodness-of-fit test results for the combination of an early exponential growth curve with a generic growth curve (fitted to daily PCR-confirmed positives) and logistic regression models (fitted to daily numbers of recoveries and deaths) using West African COVID-19 data from 28 February to 31 August 2020.

Data	Goodness-of-Fit			Overall Significance			
	DS	DF	*p*-Value	$r_{\mathrm{dev}}^{2}$ (%)	LRS	DF	*p*-Value
Reported cases	173.04	179	0.6115	95.26	3601.57	6	<0.001
Recoveries	45,028.51	184	<0.001	9.25	4861.55	1	<0.001
Deaths	499.13	184	<0.001	49.08	486.40	1	<0.001
Overall	45,700.68	175	<0.001	11.60	8949.52	8	<0.001

Table notes: DS, Deviance statistic; DF, Degrees of freedom; $r_{\mathrm{dev}}^{2}$, adjusted-deviance reduction ratio; LRS, Likelihood Ratio Statistic.

**Table 5:** Estimate, standard error (SE), Wald test statistic (z-value), *p*-value (P(>|z|)) and 95% confidence interval ($CI_{95\%}$) for the parameters of the combination of an early exponential growth curve with a generic growth curve (fitted to daily PCR-confirmed positives) and logistic regression parameters (fitted to daily numbers of recoveries and deaths) using West African COVID-19 data from 28 February to 31 August 2020.

Parameter	Estimate	SE	z-Value *	P(>|z|)	${\mathrm{CI}}_{95\%}$
$t_{e}$ (day)	29.4781	1.2368	80.1417	<0.001	[26.9413, 31.7865]
Ω (ind.)	191,290.8	6444.5420	360.9696	<0.001	[178,756.4, 204,008.2]
ω (${day}^{-1}$)	0.0148	0.0007	−87.1715	<0.001	[0.0134, 0.0162]
ν	3.7640	0.5280	9.3782	<0.001	[2.7685, 4.8240]
ρ	0.1202	0.0169	−15.1710	<0.001	[0.0884, 0.1541]
τ(day)	171.3210	2.4252	70.6431	<0.001	[166.5678, 176.0742]
σ (logind.)	0.3962	0.0201	−18.4774	<0.001	[0.3572, 0.4361]
$\kappa_{0}$	−4.0609	0.0122	−333.6829	<0.001	[−4.0848, −4.0370]
κ	0.0059	0.0001	68.5372	<0.001	[0.0058, 0.0061]
$\lambda_{0}$	−5.7136	0.0682	−83.7346	<0.001	[−5.8473, −5.5799]
λ	−0.0126	0.0006	−22.4195	<0.001	[−0.0137, −0.0115]
$\omega_{o}$ (${day}^{-1}$)	0.1660	0.0011	−261.8024	<0.001	[0.1659, 0.1662]
$\tau_{0}$ (day)	−7.2208	0.0226	−319.1971	<0.001	[−7.2651, −7.1764]
ξ (ind.)	200.3128	2.7771	382.2758	<0.001	[194.8864, 205.7716]

Table notes: ind., individuals; $t_{e}$ (day) is the duration of the exponential growth phase after the outbreak; Ω and ξ (ind.) determine the ultimate epidemic size (detected) as ξ+Ω; ω>0 (day^−1^) is the “intrinsic” growth rate constant for the sub-exponential growth phase; ν>0 is a growth acceleration parameter, ρ is a shape parameter controlling the skewness of the growth curve during the sub-exponential growth phase; τ (day) is a constant of integration determined by the initial conditions of the epidemic outbreak; σ is the logarithmic-scale standard deviation of the log-normal distribution fitted to the daily new positive case reporting data; $\kappa_{0}$ and κ are the logit-scale intercept and slope for the daily probability $\alpha_{t}$ that an active case recovers at time *t* ($\alpha_{t}=1/\left(1+e^{-(\kappa_{0}+\kappa t)}\right)$); $\lambda_{0}$ and λ are the logit-scale intercept and slope for the daily probability $\epsilon_{t}$ that an active case dies at time *t* ($\epsilon_{t}=1/\left(1+e^{-(\lambda_{0}+\lambda t)}\right)$); $\omega_{0}$ (day^−1^), $\tau_{0}$ (day) and ξ (individuals) are not free parameters, but computed using Equations (4) and (5); $\omega_{0}$ is the growth rate during the exponential growth phase; $\tau_{0}$ and ξ ensure that the daily number of positives ${\dot{C}}_{t}$ and the cumulative number of positives $C_{t}$ are smooth at $t=t_{e}$; * z-value was computed at logarithmic scale for positive definite parameters ($t_{e}$, Ω, ω, ν, ρ, σ and $\omega_{0}$), so that a *p*-value <0.05 indicates significant difference from 1 at 5% level.

**Table 6:** Estimate, standard error (SE) and 95% confidence interval ($CI_{95\%}$) for some quantities using the West African COVID-19 data from 28 February to 31 August 2020.

Quantity	Observed Value	Estimate	SE	${\mathrm{CI}}_{95\%}$
$t_{o}$ (day)	-	24.78	2.55	[19.91, 29.87]
$R_{o}$	-	2.66	0.11	[2.60, 2.69]
$I_{0}$ (ind.)	-	61.17	6.94	[47.98, 75.05]
$R_{0}$	-	2.52	0.12	[2.29, 2.76]
$t_{p}$ (day)	148	138.87	2.26	[134.45, 143.31]
${\dot{C}}_{p}$ (ind.)	2626	1804.90	83.40	[1643.19, 1969.86]
$t_{new}$ (day)	-	131.12	2.53	[126.18, 136.11]
${\dot{T}}_{max}$ (ind.)	-	22,352.97	1067.46	[20,284.04, 24,464.98]
$t_{Q_{max}}$ (day)	143	149.67	1.78	[146.18, 153.17]
$Q_{max}$ (ind.)	41,435	42,507.01	1449.81	[39,687.48, 45,368.24]
$R_{186}$ (ind.)	-	1,754,698.5	40,665.66	[1,675,407.60, 1,834,783.00]

Table notes: $t_{o}$, duration of the epidemic latency period; $R_{o}$, basic reproduction number; $I_{0}$, number of infectives at outbreak; $R_{0}$, reproduction number at outbreak; $t_{p}$, time of the peak of positive cases; ${\dot{C}}_{p}$, size of the peak of positive cases; $t_{new}$, time of the peak of new infections; ${\dot{T}}_{max}$, size of the peak of new infections; $t_{Q_{max}}$, time of the peak of active cases; $Q_{max}$, size of the peak of active cases; $R_{186}$, total number of recovered in the population at t=186 days (i.e., at the end of the studied period (31 August 2020)); - indicates not applicable.

## Data Availability

The authors confirm that the data supporting the findings of this work are available within the article.

## Abbreviations

The following abbreviations are used in this manuscript:

SIQR	Susceptible, Infective, Quarantined, Recovered model
Gen	Generic growth model
BR	Bertalanffy–Richards growth model
HG	Hyper-Gompertz growth model
Gom	Gompertz growth model
pdf	probability density function
pmf	probability mass function
LR	Likelihood Ratio
LRS	Likelihood Ratio Statistic
DS	Deviance Statistic
AIC	Akaike’s Information Criterion
ind.	Individuals
SE	Standard Error
CI	Confidence Interval
PI	Prediction Interval

## Appendix A. Generic Growth Curve and Its Limiting Cases

### Appendix A.1. Size, Rate and Acceleration

This appendix gives the population size, the growth rate and the growth acceleration of the generic growth model [8] and its limiting cases (Table A1).

**Table A1:** Generic growth model [8] and its limiting cases: population size ($\varphi_{t}$), growth rate (${\dot{\varphi }}_{t}$), and growth acceleration (${\ddot{\varphi }}_{t}$).

Model	Population Size	Growth Rate	Growth Acceleration
Gen	$\Omega {\left(1+u_{t}\right)}^{-1/\nu }$	$\Omega \omega u_{t}^{1+\rho }{\left(1+u_{t}\right)}^{-\frac{\nu +1}{\nu }}$	$\nu \omega u_{t}^{\rho }\left(\frac{\nu +1}{\nu }\frac{u_{t}}{1+u_{t}}-\rho -1\right){\dot{\varphi }}_{t}$
BR	$\Omega {\left(1+e^{-v_{t}}\right)}^{-1/\nu }$	$\Omega \omega e^{-v_{t}}{\left(1+e^{-v_{t}}\right)}^{-\frac{\nu +1}{\nu }}$	$\nu \omega \left(\frac{\nu +1}{\nu }\frac{e^{-v_{t}}}{1+e^{-v_{t}}}-1\right){\dot{\varphi }}_{t}$
HG	$\Omega \exp\left(-w_{t}^{-\frac{1}{\rho }}\right)$	$\Omega \tilde{\omega }w_{t}^{-\frac{1+\rho }{\rho }}\exp\left(-w_{t}^{-\frac{1}{\rho }}\right)$	$\tilde{\omega }w_{t}^{-1}\left(w_{t}^{-\frac{1}{\rho }}-\rho -1\right){\dot{\varphi }}_{t}$
Gom	$\Omega \exp\left(-e^{-x_{t}}\right)$	$\Omega \tilde{\omega }\exp\left(-x_{t}-e^{-x_{t}}\right)$	$\tilde{\omega }\left(e^{-x_{t}}-1\right){\dot{\varphi }}_{t}$

Table notes: Gen, Generic; BR, Bertalanffy–Richards; HG, Hyper-Gompertz; Gom, Gompertz; $u_{t}={\left[1+\omega \nu \rho (t-\tau )\right]}^{-1/\rho }$, $v_{t}=\nu \omega \left(t-\tau \right)$, $w_{t}=\tilde{\omega }\rho \left(t-\tau \right)$ and $x_{t}=\tilde{\omega }\left(t-\tau \right)$.

The restriction $-1<\rho <\nu^{-1}$ given in Section 2.1 makes the parameters ν and ρ dependent. This can be circumvented by introducing a free working shape parameter $\rho_{0}\in \left(0,1\right)$ such that $\rho =(\rho_{0}\left(\nu +1\right)/\nu )-1$ [5].

### Appendix A.2. Peak Time and Size

This appendix gives the peak (time and size) related to the generic growth model [8] and its limiting cases (Table A2).

**Table A2:** Peak time ($t_{p}$) and size (${\dot{\varphi }}_{p}={\dot{\varphi }}_{t_{p}}$) of the generic growth curve [8] and its limiting cases.

Model	Peak Time ($t_{p}$)	Peak Size (${\dot{\varphi }}_{p}$)
Generic	$\tau +\frac{1}{\nu \omega \rho }\left\{{\left[\frac{1-\nu \rho }{\nu (1+\rho )}\right]}^{\rho }-1\right\}$	$\Omega \omega {\left[\nu \frac{1+\rho }{1-\rho \nu }\right]}^{1+\rho }{\left(\frac{\nu +1}{1-\rho \nu }\right)}^{-\frac{\nu +1}{\nu }}$
BR (ρ→0)	$\tau -\frac{\log\nu }{\omega \nu }$	$\Omega \omega \nu {\left(1+\nu \right)}^{-\frac{\nu +1}{\nu }}$
HG (ν→0, $\nu \omega^{\left(1+\rho \right)}\to \tilde{\omega }$)	$\tau +\frac{{\left(1+\rho \right)}^{-\rho }}{\tilde{\omega }\rho }$	$\Omega \tilde{\omega }{\left[\left(1+\rho \right)e^{-1}\right]}^{1+\rho }$
Gompertz (ρ→0 in HG)	τ	$\Omega \tilde{\omega }e^{-1}$

Table notes: BR, Bertalanffy–Richards; HG, Hyper-Gompertz; $u_{p}=\nu \left(1+\rho \right)/\left(1-\rho \nu \right)$, $t_{p}$ is the root of ${\ddot{\varphi }}_{t}$; the expressions of ${\dot{\varphi }}_{t}$ (growth rate) and ${\ddot{\varphi }}_{t}$ (growth acceleration) are given in Table A1.

## Appendix B. Dynamics of Detected and Active Cases

Based on the the recovery rate $\alpha_{t}$ and the death rate $\epsilon_{t}$ given in (11) and (12), at the outbreak (t=0), the recovery rate is $\alpha_{0}=1/\left(1+e^{-\kappa_{0}}\right)$ and the death rate is $\epsilon_{0}=1/\left(1+e^{-\lambda_{0}}\right)$. Then, along the epidemic course, κ and λ determine the changes in the log-odds ratio to have an outcome per unit time. Under constant removal rates assumption (κ=λ=0), solving the differential (10), gives the actives cases as (assuming that $C_{w}$ is differentiable for 0<w<t)

(A1) $$\begin{array}{ccc} Q_{t} & = & \left[Q_{0}+\int_{0}^{t}{\dot{C}}_{w}e^{(\alpha_{0}+\epsilon_{0})w}dw\right]e^{-(\alpha_{0}+\epsilon_{0})t}. \end{array}$$

Taking the expression of $C_{t}$ in (1) into account yields for κ=λ=0

(A2) $$\begin{array}{ccc} Q_{t} & = & \left\{\begin{array}{cc} Q_{0}e^{-(\alpha_{0}+\epsilon_{0})t}+\frac{\delta I_{0}}{\omega_{0}+\alpha_{0}+\epsilon_{0}}\left[e^{\omega_{0}t}-e^{-(\alpha_{0}+\epsilon_{0})t}\right]\; & \;\;\;\;\;\mathrm{if}\;\;0<t\leq t_{e}\\ \left[Q_{e}e^{\left(\alpha_{0}+\epsilon_{0}\right)t_{e}}+\int_{t_{e}}^{t}{\dot{\varphi }}_{r}e^{(\alpha_{0}+\epsilon_{0})r}dr\right]e^{-(\alpha_{0}+\epsilon_{0})t}\; & \mathrm{if}\;\;t>t_{e} \end{array}\right.\;\;\;\;\; \end{array}$$

where $Q_{e}=Q_{t_{e}}$ is the number of active cases at the end of the exponential growth phase. For the general situation where the rates $\alpha_{t}$ and $\epsilon_{t}$ may be time dependent, the number of active cases is given by (13) in accordance with (A1).

## Appendix C. Overall Epidemic Dynamics

### Appendix C.1. The SIQR Model

Hethcote et al. [22] showed in the case of a constant transmission rate ($\beta_{t}=\beta$) that the system can have an endemic equilibrium point. Furthermore, such endemic points may be either locally asymptotically stable or subject to Hopf bifurcation depending on model parameters, giving rise to unstable spiral and periodic solutions [22]. In the modeling framework considered in this work, the long-term dynamics of a target disease is solely determined by the ultimate epidemic size Ω (detected). Indeed, $\lim_{t\to \infty }\varphi_{t}=\Omega$ so that $\lim_{t\to \infty }{\dot{\varphi }}_{t}=0$ since Ω is finite and, therefore, $\lim_{t\to \infty }I_{t}=0$ by (8). Consequently, the disease always dies out in the long run and the system tends to the disease-free equilibrium $P_{0}$. This happens because the fraction of infectives in the population decreases to very near zero and the fraction of quarantined (*Q*) decreases to zero (through recovery and death). Eventually, over 100 or more years, the recovered people (*R*) slowly die off and the birth process slowly increases the susceptibles (*S*), until everyone is susceptible at the disease-free equilibrium $P_{0}$ [55]. Note, however, that the SIQR model described by (16)–(19) together with (8) is meant for a single epidemic wave, whereas it is possible to have successive epidemic waves or even overlapping epidemic waves [1] which would be described by a mixture of many SIQR models.

From (18), the number $Q_{t}$ of known active cases before the outbreak, is given by

(A3) $$\begin{array}{c} Q_{t}=Q_{o}e^{-\left(\alpha_{0}+\epsilon_{0}\right)\left(t+t_{o}\right)}\;\;\;\mathrm{for}\;\;-t_{o}\leq t\leq 0 \end{array}$$

on assuming constant recovery ($\alpha_{0}$) and death ($\epsilon_{0}$) rates before the outbreak and on denoting $Q_{o}$ the number of persistent cases from previous epidemic waves (e.g., $Q_{o}=0$ for a new disease-related epidemic).

### Appendix C.2. Susceptibles, Recovered, Total and Lost Cases

In addition to infectives ($I_{t}$) and actives ($Q_{t}$) already available from the growth curve $C_{t}$, the computation of the population size in (15) requires the expressions of the sizes of the compartments of susceptibles ($S_{t}$) and immunes ($R_{t}$). Inserting Equation (20) into (16) and replacing in light of (8) ${\dot{I}}_{t}/I_{t}=\omega_{0}$ for t≤0 and ${\dot{I}}_{t}/I_{t}={\ddot{C}}_{t}/{\dot{C}}_{t}$ for t>0 yields

(A4) $$\begin{array}{ccc} {\dot{S}}_{t} & = & \left\{\begin{array}{cc} \eta -\left(\gamma +\pi +\omega_{0}\right)I_{0}e^{\omega_{0}t}-\mu S_{t}\; & \mathrm{if}\;\;t\leq 0\\ \eta -\delta^{-1}\left[\left(\gamma +\delta +\pi \right){\dot{C}}_{t}+{\ddot{C}}_{t}\right]-\mu S_{t}\; & \mathrm{if}\;\;t>0 \end{array}\right..\;\;\;\; \end{array}$$

Therefore, the number of susceptible individuals is given for $-t_{o}\leq t\leq 0$ by

(A5) $$\begin{array}{ccc} S_{t} & = & \frac{\eta }{\mu }+\left(S_{o}-\frac{\eta }{\mu }\right)e^{-\mu (t_{o}+t)}-\frac{\omega_{0}+\gamma +\pi }{\omega_{0}+\mu }I_{0}\left(e^{\omega_{0}t}-e^{-\left(\omega_{0}+\mu \right)t_{o}-\mu t}\right) \end{array}$$

where $S_{o}$ is the number of susceptibles at the beginning of the epidemic, obtained from (15) with $t=-t_{o}$ ($I_{o}=1$) as

(A6) $$\begin{array}{c} S_{o}=N_{o}-Q_{o}-R_{o}-1 \end{array}$$

where $N_{o}$ is the initial population size (i.e., at $t=-t_{o}$) and $K_{o}$ is the number of known immune individuals at the beginning of the target epidemic (recovered from past outbreaks if any). The number of susceptibles after the outbreak (t>0) is

(A7) $$\begin{array}{ccc} S_{t} & = & \left\{\begin{array}{c} \frac{\eta }{\mu }+\left(S_{0}-\frac{\eta }{\mu }\right)e^{-\mu t}-\frac{\omega_{0}+\gamma +\delta +\pi }{\omega_{0}+\mu }I_{0}\left(e^{\omega_{0}t}-e^{-\mu t}\right)\;\;\;\;\;\;\;\;\;\;\;\;\;\;\;\;\;\;\mathrm{if}\;\;0<t\leq t_{e}\;\;\;\;\;\;\\ \frac{\eta }{\mu }+\left(S_{e}-\frac{\eta }{\mu }\right)e^{\mu (t_{e}-t)}-\left\{\int_{t_{e}}^{t}\left[1+\delta^{-1}\left(\gamma +\pi +z_{r}\right)\right]{\dot{\varphi }}_{r}e^{\mu r}dr\right\}e^{-\mu t}\;\mathrm{if}\;t>t_{e}\;\;\;\;\; \end{array}\right. \end{array}$$

where $S_{0}$ is the number of susceptibles at the outbreak (t=0) and is available from (A5), $S_{e}=S_{t_{e}}$ is the number of susceptibles at the end of the exponential growth phase and $z_{t}={\ddot{\varphi }}_{t}/{\dot{\varphi }}_{t}$ is the ratio of the growth acceleration ${\ddot{\varphi }}_{t}$ to the growth rate ${\dot{\varphi }}_{t}$ (Table 2).

From the transfer diagram in Figure 1, the total number of individuals who were infected and then recovered, and are alive can be decomposed as

(A8) $$\begin{array}{c} R_{t}=K_{t}+U_{t} \end{array}$$

where $K_{t}$ is the number of individuals who were tested positive, were isolated and then recovered (known) and $U_{t}$ is the number of individuals who contracted the infection but were not detected and have recovered (unknown). Equation (19) is then equivalent to the system

(A9) $$\begin{array}{ccc} {\dot{K}}_{t} & = & \alpha_{t}Q_{t}-\mu K_{t} \end{array}$$

(A10) $$\begin{array}{ccc} {\dot{U}}_{t} & = & \gamma I_{t}-\mu U_{t}. \end{array}$$

From (A9), the number of known recovered individuals $K_{t}$ is given for $-t_{o}\leq t\leq 0$ by

(A11) $$\begin{array}{ccc} K_{t} & = & \left\{\begin{array}{cc} \left[K_{o}+\alpha_{0}Q_{o}\left(t_{o}+t\right)\right]e^{-\mu (t_{o}+t)}\; & \mathrm{if}\;\mu =\alpha_{0}+\epsilon_{0}\\ K_{o}e^{-\mu (t_{o}+t)}+\frac{\alpha_{0}Q_{o}}{\mu -(\alpha_{0}+\epsilon_{0})}\left[e^{-\left(\alpha_{0}+\epsilon_{0}\right)\left(t_{o}+t\right)}-e^{-\mu (t_{o}+t)}\right]\; & \mathrm{if}\;\mu \neq \alpha_{0}+\epsilon_{0} \end{array}\right..\;\;\;\;\; \end{array}$$

After the outbreak, $K_{t}$ is given by

(A12) $$\begin{array}{ccc} K_{t} & = & \left\{\begin{array}{cc} \left[K_{0}+\int_{0}^{t}\alpha_{r}Q_{r}e^{\mu r}dr\right]e^{-\mu t}\; & \;\;\;\;\;\mathrm{if}\;\;0<t\leq t_{e}\\ \left[K_{e}e^{\mu t_{e}}+\int_{t_{e}}^{t}\alpha_{r}Q_{r}e^{\mu r}dr\right]e^{-\mu t}\; & \mathrm{if}\;\;t>t_{e} \end{array}\right. \end{array}$$

where $K_{0}$ (available from (A11)) is the number of known recovered individuals before the considered outbreak (recovered from past outbreaks if any) and $K_{e}=K_{t_{e}}$ is the number of known recovered individuals at the end of the exponential growth phase. From (A10), the number of unknown recovered individuals is

(A13) $$\begin{array}{ccc} U_{t} & = & \left\{\begin{array}{cc} \frac{\gamma }{\omega_{0}+\mu }I_{0}\left[e^{\omega_{0}t}-e^{-\left(\omega_{0}+\mu \right)t_{o}-\mu t}\right]\; & \;\;\;\;\;\;\;\;\mathrm{if}\;-t_{o}\leq t\leq t_{e}\\ \left[U_{e}e^{\mu t_{e}}+\gamma \delta^{-1}\int_{t_{e}}^{t}{\dot{\varphi }}_{r}e^{\mu r}dr\right]e^{-\mu t}\; & \mathrm{if}\;t>t_{e} \end{array}\right. \end{array}$$

where $U_{e}=U_{t_{e}}$ is the number of undetected and recovered cases at the end of the exponential growth phase.

The total number of persons infected during an epidemic wave is indicative of the overall cost of the epidemic in terms of its overall impact on the society (in regard to, e.g., health, work and communication). The total number of new infections denoted ${\dot{T}}_{t}$ is given by

(A14) $$\begin{array}{c} {\dot{T}}_{t}=\left(\gamma +\delta_{t}+\pi \right)I_{t}+{\dot{I}}_{t}. \end{array}$$

The total number of cases is thus given by

(A15) $$\begin{array}{ccc} T_{t} & = & \left\{\begin{array}{cc} 1+\frac{\omega_{0}+\gamma +\pi }{\omega_{0}}\left(I_{0}e^{\omega_{0}t}-1\right)\; & \;\;\;\;\;\;\;\mathrm{if}\;\;-t_{o}\leq t\leq 0\\ T_{0}+\frac{\omega_{0}+\gamma +\delta +\pi }{\omega_{0}}I_{0}\left(e^{\omega_{0}t}-1\right)\; & \;\;\;\;\;\mathrm{if}\;\;0<t\leq t_{e}\\ T_{e}+\delta^{-1}\left[\left(\gamma +\delta +\pi \right)\left(\varphi_{t}-\varphi_{e}\right)+{\dot{\varphi }}_{t}-{\dot{\varphi }}_{e}\right]\; & \mathrm{if}\;\;t>t_{e} \end{array}\right.\;\;\;\;\; \end{array}$$

where $T_{e}=T_{t_{e}}$, $\varphi_{e}=\varphi_{t_{e}}$ and ${\dot{\varphi }}_{e}={\dot{\varphi }}_{t_{e}}$. The increase in lost cases is ${\dot{\Lambda }}_{t}=\left(\gamma +\pi \right)I_{t}$ per unit time so that the number of lost cases $\Lambda_{t}$ is given by

(A16) $$\begin{array}{ccc} \Lambda_{t} & = & \left\{\begin{array}{cc} \frac{\gamma +\pi }{\omega_{0}}\left(I_{0}e^{\omega_{0}t}-1\right)\; & \;\;\;\;\;\;\;\;\;\mathrm{if}\;\;-t_{o}\leq t\leq t_{e}\\ \Lambda_{e}+\frac{\gamma +\pi }{\delta }\left(\varphi_{t}-\varphi_{e}\right)\; & \mathrm{if}\;\;t>t_{e} \end{array}\right.. \end{array}$$

with $\Lambda_{e}=\Lambda_{t_{e}}$. In particular, the number of lost cases during the entire epidemic latency period is $\Lambda_{0}=\left(\gamma +\pi \right)\left(I_{0}-1\right)/\omega_{0}$.

### Appendix C.3. Epidemic Peak

The peak of new infections occurs when ${\ddot{T}}_{t}$ vanishes. We have from (A14)

(A17) $$\begin{array}{c} {\ddot{T}}_{t}=\left(\gamma +\delta_{t}+\pi \right){\dot{I}}_{t}+{\ddot{I}}_{t}. \end{array}$$

During the exponential growth phase, both ${\dot{I}}_{t}$ and ${\ddot{I}}_{t}$ are increasing functions of time so that the peak of new infections occurs after $t_{e}$, i.e., the peak time $t_{new}$ satisfies $t_{new}>t_{e}$. Hence, the peak time is the solution of

(A18) $$\begin{array}{c} \left(\gamma +\delta +\pi \right){\ddot{\varphi }}_{t}+{\dddot{\varphi }}_{t}=0 \end{array}$$

which can be solved for *t* using a numerical root finding routine such as the R [24] function *uniroot* or the Matlab [25] function *fzero*. Afterwards, the peak size ${\dot{T}}_{new}$ (the maximum number of new infections per unit time) is obtained by inserting $t_{new}$ in (A14).

### Appendix C.4. Long-Term Epidemic Dynamics

The specification of the growth model in (1) to an epidemic implicitly assumes that the number of infectives in (8) peaks at time $t_{p}$ and then tends to zero. The decay of infectives after the peak can happen at various rates, depending on the growth pattern (determined by contacts between infectives and susceptibles or intermediate hosts), the response of infected individuals’s organism (natural or induced with medicine or a vaccine) to the disease (recovery and death process) and the testing efforts (detection followed by isolation). There are actually two alternative paths from a disease related state (i.e., $I_{t}>0$) toward the unique (disease-free) equilibrium $P_{0}$: transmissions either stop ($R_{t}$ reaches zero) or continue for a long time at a rate which cannot sustain an epidemic ($0<R_{t}\leq 1$). We discuss these two scenarios in this section. Because the behavior of $R_{t}$ for $t>t_{e}$ depends on $z_{t}={\ddot{\varphi }}_{t}/{\dot{\varphi }}_{t}$ (see (24)), we make use of the minimum of $z_{t}$ (over $t>t_{e}$) and the limit $\lim_{t\to \infty }z_{t}$ given in Table A3 for the general and limiting expressions of $z_{t}$.

**Table A3:** Minimum point ($t_{z_{min}}=\underset{t>t_{e}}{\arg}\min\left\{z_{t}\right\}$), minimum value $z_{min}=\min_{t>t_{e}}\left\{z_{t}\right\}$ and limit $z_{lim}=\lim_{t\to \infty }z_{t}$ of the ratio $z_{t}={\ddot{\varphi }}_{t}/{\dot{\varphi }}_{t}$ of the growth acceleration ${\ddot{\varphi }}_{t}$ to the growth rate ${\dot{\varphi }}_{t}$ of the generic growth curve ($\varphi_{t}$) [8] and its limiting cases

Model	$t_{z_{\min}}$	$z_{\min}$	$z_{\lim}$
Generic	$\tau +\frac{1}{\nu \omega \rho }\left(u_{z}^{-\rho }-1\right)$	$\nu \omega u_{z}^{\rho }\left(\frac{\nu +1}{\nu }\frac{u_{z}}{1+u_{z}}-\left(1+\rho \right)\right)$	0
BR (ρ→0)	*∞*	−νω	−νω
HG (ν→0, $\nu \omega^{\left(1+\rho \right)}\to \tilde{\omega }$)	$\tau +{\tilde{\omega }}^{-1}\rho^{-(1+\rho )}$	$-\tilde{\omega }\rho^{\rho }$	0
Gompertz (ρ→0 in HG)	*∞*	$-\tilde{\omega }$	$-\tilde{\omega }$

Table notes: BR, Bertalanffy–Richards; HG, Hyper-Gompertz; $\varphi_{t}$ is as defined in (2) and $z_{t}$ is available from Table A1, $u_{z}=\left(\sqrt{1-\rho_{0}}-1\right)/\sqrt{1-\rho_{0}}$ with $\rho_{0}=\nu \left(\rho +1\right)/\left(\nu +1\right)$.

#### Appendix C.4.1. Straight End of Transmissions

The transmission of a target disease ends when the transmission rate $\beta_{t}$ and accordingly the number of new infections (${\dot{T}}_{t}$) drops to zero at a finite time point which is the solution to the equation

(A19) $$\begin{array}{c} z_{t}+\left(\gamma +\delta +\pi \right)=0. \end{array}$$

Actually, because the transmission rate per capita per unit time $\beta_{t}\left(S_{t}+R_{t}\right)/\left(N_{t}-Q_{t}\right)$ is a non-negative quantity, (20) implicitly assumes that ${\dot{I}}_{t}/I_{t}\geq -\left(\gamma +\delta_{t}+\pi \right)$. This condition holds for $t\leq t_{e}$ since ${\dot{I}}_{t}/I_{t}=\omega_{0}>0$. For the sub-exponential growth phase ($t>t_{e}$), the assumption is equivalent to

(A20) $$\begin{array}{c} z_{t}+\left(\gamma +\delta +\pi \right)\geq 0. \end{array}$$

The importance of the inequality in (A20) becomes more apparent when considering the reproduction number given in (24): the restriction ensures that $R_{t}\geq 0$. Therefore, if (A19) has a solution $t_{z}\in \left(t_{new},\infty \right)$, then the transmission of the infection (from the infectives already present in the population to the susceptibles) ends at $t=t_{z}$ and $R_{t}=0$ for $t\geq t_{z}$. The existence of a solution $t_{z}$ of (A19) can be checked by comparing the minimum value $z_{min}$ of $z_{t}$ (Table A3) to the total rate (γ+δ+π) of removals from $I_{t}$. Indeed, if we have $z_{min}=-\left(\gamma +\delta +\pi \right)$, then $t_{z}=t_{min}$. Furthermore, if $z_{min}<-\left(\gamma +\delta +\pi \right)$, there exists a solution $t_{z}\in \left(t_{new},t_{min}\right)$ which can be found using a numerical routine. In either of these two cases, the number of susceptibles afterwards stays at $S_{z}=S_{t_{z}}$ and the number of infectives follows an exponential decay as

(A21) $$\begin{array}{c} I_{t}=I_{z}\;e^{-\left(\gamma +\delta +\pi \right)\left(t-t_{z}\right)}\;\;\;\mathrm{for}\;\;t>t_{z} \end{array}$$

where $I_{z}=I_{t_{z}}$ is given by (8). The number of new detected cases is $\dot{C_{t}}=\delta I_{t}$ as before, but the number of known active cases becomes

(A22) $$\begin{array}{c} Q_{t}=\left[Q_{z}F_{z}+\delta I_{z}\int_{t_{z}}^{t}e^{-(\gamma +\delta +\pi )(r-t)}F_{r}dr\right]F_{t}^{-1}. \end{array}$$

where $Q_{z}=Q_{t_{z}}$ is given by (13) and $F_{z}=F_{t_{z}}$ is given by (14). Whereas the number $K_{t}$ of known immunes has the same expression given in (A12) with $Q_{t}$ given by (A22), the number $U_{t}$ of unknown immunes becomes

(A23) $$\begin{array}{c} U_{t}=U_{z}\;e^{-\mu (t-t_{z})}-\frac{\gamma I_{z}}{\gamma +\delta +\pi -\mu }\left[e^{-\left(\gamma +\delta +\pi \right)\left(t-t_{z}\right)}-e^{-\mu (t-t_{z})}\right] \end{array}$$

where $U_{z}=U_{t_{z}}$ is given by (A13). From (A21), the number of infectives falls to 1 at time

(A24) $$\begin{array}{c} t_{f}=t_{z}+\frac{\log I_{z}}{\gamma +\delta +\pi }. \end{array}$$

Finally, since the removal rate of infectives is γ+δ+π per unit time, the probability that the number of infectives drops to zero at a time $t_{end}=t_{f}+r$ with *r* a non-negative integer is $\left(\gamma +\delta +\pi \right){\left(1-\gamma -\delta -\pi \right)}^{r}$. Under this scenario, the system (16)–(19) will tend to the disease free equilibrium $P_{0}$ at which the size of the population stabilizes at $N^{*}=\eta /\mu$.

#### Appendix C.4.2. Asymptotic End of Transmissions

When the shape of the curve of infectives has growth parameters such that $z_{t}={\ddot{\varphi }}_{t}/{\dot{\varphi }}_{t}>-\left(\gamma +\delta +\pi \right)$ for $t>t_{e}$, the transmission of the disease does not stop straightly, but continues at a low rate. Indeed, under this scenario, inserting the limit $\lim_{t\to \infty }I_{t}=0$ in (24) yields

(A25) $$\begin{array}{c} R_{\infty }=\lim_{t\to \infty }R_{t}=\left(1+\frac{z_{lim}}{\gamma +\delta +\pi }\right) \end{array}$$

where $z_{lim}=\lim_{t\to \infty }z_{t}\leq 0$ is available in Table A3 (note that $z_{lim}=-\nu \omega$ when ρ→0 and $z_{lim}=0$ otherwise). Therefore, $R_{\infty }\leq 1$ and the population asymptotically tends to the disease-free equilibrium $P_{0}$ [22]. However, if $z_{lim}>-\left(\gamma +\delta +\pi \right)$, then we also have $R_{\infty }>0$. For instance, under the simple logistic growth model (ν=1, ρ→0), $z_{t}$ decreases and tends to −ω as t→∞ (Table A3) and $R_{\infty }=1-\omega /\left(\gamma +\delta +\pi \right)$ which satisfies $0<R_{\infty }<1$ (from ω<γ+δ+π). In general, when ρ≠0, the shape of $\varphi_{t}$ may allow $z_{t}$ to properly decrease for $t>t_{e}$ and become negative from $t>t_{new}$ so that $R_{t}<1$. However, when $z_{t}$ reaches its limit $z_{lim}>-\left(\gamma +\delta +\pi \right)$, it bounces and tends to 0 (Table A3) so that $R_{\infty }=1$.

The limit (A25) shows that, when $R_{t}$ does not sharply reach zero but ρ→0, the asymptotic reproduction number depends on rate parameters (γ, δ and π) that can be controlled to hasten the disease to die out. In the situation, where ρ≠0, $R_{\infty }$ is independent of model parameters, so that the long run dynamics is less likely to respond to changes in the rate parameters.

## Appendix D. Goodness-of-fit and Model Selection

We define the likelihood $\ell_{s}$ of the saturated model by replacing ${\dot{C}}_{t}$ in (26) by the observed values $Y_{t}$, $\alpha_{t}$ in (27) by the observed daily recovery probabilities $G_{t}/\left(Q_{t-1}+Y_{t}\right)$ and $\epsilon_{t}$ in (28) by the observed daily death probabilities $M_{t}/\left(Q_{t-1}+Y_{t}\right)$. Similarly, we define the likelihood $\ell_{n}$ of the null model by replacing each ${\dot{C}}_{t}$ by the daily mean count $\bar{Y}=n^{-1}\sum_{t=1}^{n}Y_{t}$, each $\alpha_{t}$ by the overall daily recovery probability $\bar{\alpha }$ (obtained assuming κ=0) and each $\epsilon_{t}$ by the overall daily death probability $\bar{\epsilon }$ (obtained assuming λ=0). The residual deviance of the maximum likelihood fit is then given by $DEV_{res}=2\left(\ell_{s}-\ell \left(\hat{\theta }\right)\right)$ and the null deviance of the null model fit is given by $DEV_{null}=2\left(\ell_{s}-\ell_{n}\right)$. The quantity $DEV_{res}$ is a statistic to test the null hypothesis $H_{0}$: the assumed model is not significantly different from the unknown model that generated the data. If $H_{0}$ is true, then the large sample distribution (i.e., as n→∞) of $DEV_{res}$ is the $\chi_{k}^{2}$ distribution with k=n−m degrees of freedom where m=12 is the number of individual model parameters in θ [56]. If the overall goodness of fit test based on $DEV_{res}$ rejects $H_{0}$, then the corresponding statistics (residual deviances) can be computed for the three sub-models (i.e., considering the log-likelihoods in (29)–(32)) to identify the sub-models lacking goodness-of-fit. The percentage of information explained by the maximum likelihood fit for the cumulative data can be evaluated using the common adjusted-coefficient of determination

(A26) $$\begin{array}{c} r_{a}^{2}=1-\left(1-r^{2}\right)\frac{n-1}{n-m_{Y}} \end{array}$$

where $r=cor\left(C_{t},Y_{\;.t}\right)$ is the Pearson’s correlation coefficient between $C_{t}$ and $Y_{\;.t}=\sum_{j=1}^{t}\;Y_{j}$, and $m_{Y}$ is the number of individual model parameters in $\theta_{Y}$. The explanative power of the overall fit can be assessed via the adjusted-deviance reduction ratio [57]

(A27) $$\begin{array}{c} r_{dev}^{2}=1-\frac{DEV_{res}}{DEV_{null}}\frac{n-1}{n-m}. \end{array}$$

Let $H\;\left(\theta \right)$ be the hessian matrix of *ℓ* and define the asymptotic covariance matrix $\Sigma \left(\theta \right)=-{\left[H\left(\theta \right)\right]}^{-1}$. In a large sample, the covariance matrix of the maximum likelihood estimate $\hat{\theta }$ is estimated by $\hat{\Sigma }=\Sigma \left(\hat{\theta }\right)$ and square roots of the diagonal elements of $\hat{\Sigma }$ provide standard errors for individual parameters in θ. For the selection of the parsimonious model agreeing with the observed data, the likelihood ratio statistic can be used. To test a null hypothesis $H_{0}$ against an alternative $H_{1}$ with q>0 restrictions fewer than $H_{0}$, the likelihood ratio (LR) statistic is given by [27]

(A28) $$\begin{array}{ccc} LR & = & 2\left[\ell \left({\hat{\theta }}^{\left(1\right)}\right)-\ell \left({\hat{\theta }}^{\left(0\right)}\right)\right] \end{array}$$

where ${\hat{\theta }}^{\left(0\right)}$ is the estimate under $H_{0}$ and ${\hat{\theta }}^{\left(1\right)}$ is the estimate under $H_{1}$. If the null hypothesis $H_{0}\;$ is true, the test statistic LR converges in distribution to the $\chi_{\left(q\right)}^{2}$ distribution with *q* degrees of freedom as n→∞ [39]. There are however distinct special cases of model (2) leading to the same number of parameters (*m*). For example, we have the Bertalanffy–Richards (m=7), hyper-logistic (m=7) and hyper-Gompertz (m=7). We also have the Gompertz (m=6) and logistic (m=6). In these situations, q=0 and the likelihood ratio test cannot be used. Thereafter, we suggest to consider information criteria such as the Akaike’s Information Criterion (AIC) (the lower, the better): $\mathrm{AIC}=-2\ell (\hat{\theta })+2m$ [40].
